# Iodine(I) pnictogenate complexes as Iodination reagents

**DOI:** 10.1038/s42004-024-01240-0

**Published:** 2024-07-17

**Authors:** Sharath Mohan, Kari Rissanen, Jas S. Ward

**Affiliations:** Department of Chemistry, University of Jyvaskyla, 40014 Jyväskylä, Finland

**Keywords:** Chemical bonding, Coordination chemistry

## Abstract

Halogen(I) complexes are widely used as halogenation reagents and traditionally feature homoleptic stabilising Lewis bases, though the recent revitalisation of iodine(I) carboxylate chemistry has provided isolable examples of heteroleptic iodine(I) complexes. This work reports iodine(I) pnictogenate complexes stabilised by a Lewis base (L), Ph_2_P(O)O─I─L, synthesised via cation exchange from the silver(I) precursor, (Ph_2_P(O)OAg)_n_. The complexes were characterised in both solution (^1^H, ^1^H-^15^N HMBC, ^31^P) and the solid state, and supplemented computationally by DFT studies. Interestingly, these iodine(I) pnictogenates demonstrate a range of stabilities, and have been found to excel as iodination reagents in comparison to carbonyl hypoiodites, with comparable reactivity to the eponymous Barluenga’s reagent in the iodination of antipyrine.

## Abstract (web summary)

Iodine(I) carboxylates have been explored as iodination reagents, but the role of the carbonyl group in promoting such reactivity remains poorly understood. Here, the authors prepare iodine(I) pnictogenates and find that they excel as iodination reagents in comparison to iodine(I) carboxylates.

## Introduction

The utility of halogen bonding as a productive non-covalent interaction has been realised over the last few decades, with applications in a myriad of host–guest systems (e.g., chemical or biomolecular separations) and the preparation of functional materials (e.g., liquid-crystalline, magnetic, phosphorescent, porous)^1–4^. Halogen bonding, defined as the interaction between an electrophilic region of a halogen atom with neutral or anionic nucleophiles^5^, is also adept toward the assembly of supramolecular architectures^6–11^, largely owing to its highly directional bonding. This is especially true for the sub-class of halogen bonded species, halogen(I) (halenium) ions, which are fully polarised halogen atoms (X^+^; X = I, Br, Cl) that demonstrate linear 2-coordinate complexes of the form [L─X─L]^+^ in the presence of a pair of suitable Lewis bases (L)^12–18^, which have also been incorporated into supramolecular iodine(I) architectures^19–23^. Though first identified in the 1960s^24–26^, it was not until the 1980s when Barluenga and co-workers breathed new life into the study of halogen(I) complexes by demonstrating their versatility as mild iodination and oxidation reagents toward a wide variety of substrates^27–31^, which endures to this day through the continued use of *Barluenga’s reagent*, [I(pyridine)_2_]BF_4_.

Studies into the use of alkyl hypoiodites of the form R─OI (R = alkyl group) as iodination reagents also have a long history ^32–35^. As in situ generated reagents, these species have demonstrated an impressive capacity to iodinate non-activated substrates such as alkanes (e.g., conversion of n-butane to 2-iodobutane)^32^. Unlike for [N─I─N]^+^ type complexes where the structure was identified early on^24^, the composition of the actual reactive species for alkyl hypoiodites is still unclear (cf. “^t^BuOI”)^34,36^, preventing definitive structure–reactivity relationships from being established. Even the perhaps *best*-characterised carbonyl hypoiodite, CH_3_C(O)OI, had only been observed in solution by ^1^H NMR spectroscopy and spectrophotometrically ^37,38^. Whilst named as *hypoiodites* due to their inclusion of an O─I group, their reactivity as iodine(I) reagents does not reflect this term, and therefore the term *iodine(I) carboxylates* will be used henceforth. Nevertheless, the solid-state structure of CH_3_C(O)OI was later confirmed by an SCXRD study via isolation of the stabilised iodine(I) carboxylate, C(O)O─I─N, complexes using 4-substituted pyridine derivatives^39^. This overlooked ability to stabilise iodine(I) carboxylates and definitively characterise them in the solid state via SCXRD^40^, has been recently revitalised, fuelled by the observation that these charge-neutral C(O)O─I─N derivatives can themselves be considered iodine(I) species, and are therefore analogous to [N─I─N]^+^ type complexes.

**Fig. 1:**
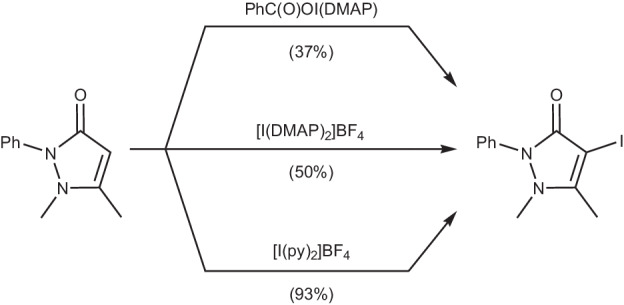
A comparison of the iodination of antipyrine using three different reagents. Reaction yields after 2 h in CH_2_Cl_2_ (average isolated percentage yields given in brackets; py = pyridine, DMAP = *N*,*N*-dimethylpyridin-4-amine) for iodine(I) carboxylate (top), its direct [N─I─N]^+^ analogue (middle), and Barluenga’s reagent (bottom)^41,62^.

The reactivity of iodine(I) carboxylate complexes as iodination reagents have been tested on antipyrine as a model substrate, though they were found to be lacklustre in comparison to [I(pyridine)_2_]BF_4_ (Fig. 1)^41^. Anionic dioxoiodane complexes, which feature an [O‒I‒O]^−^ motif, can also be seen as closely related analogues to the [N─I─N]^+^ and C(O)O‒I‒N complexes and are likewise known as organic reagents^42,43^.

To date, no solid-state examples of RO─I (R = alkyl) exist, stabilised or otherwise. This presents an interesting question as to the necessity of the carbonyl group connected to the iodine-coordinated oxygen atom, and its role in promoting O‒I‒N complex formation. This year, a novel subset of O─I─N derivatives based on sulfonates of the general form RS(O)_2_O─I─L (R = Me, 4-tolyl, C_7_H_15_; L = 4-substituted pyridine) was reported^44^, which highlighted that non-carbonyl O─I─N complexes can be isolated in the solid state. Nevertheless, their observed decomposition within seconds of being removed from their low-temperature parent reaction mixtures also supports that the carbonyl group is favourably stabilising the O─I─N complex formation, given that iodine(I) carboxylate complexes can be isolated without this same rapid decomposition. Therefore, to better explore this mystery, the pursuit of non-carbonyl O─I─N complexes was attempted based on pnictogen (P, As) alternatives, with six iodine(I) phosphinate complexes stabilised by a variety of Lewis bases being successfully synthesised.

## Results and discussion

### Synthesis

The commercially available diphenylphosphinic acid, Ph_2_P(O)OH, was seen as a pnictogen analogue of benzoic acid that had previously found success in the synthesis of iodine(I) complexes. This precursor was seen as an ideal analogue, especially given that the silver(I) derivative, (Ph_2_P(O)OAg)_*n*_ (silver(I) diphenylphosphinate), necessary for subsequent iodine(I) complex synthesis had also already been reported^45^. Nonetheless, a higher-yielding procedure for the synthesis of (Ph_2_P(O)OAg)_*n*_ (**1**) was developed, which gave the species in an 82% yield (Supplementary Note 2).

**Fig. 2:**
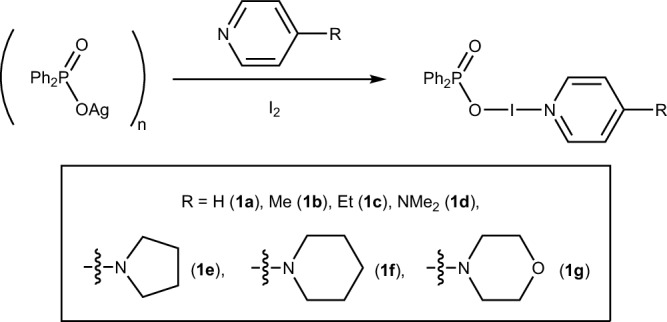
The general synthetic procedure for the synthesis of iodine(I) pnictogenate complexes. The numbering convention used for the iodine(I) pnictogenates synthesised herein.

Reaction of (Ph_2_P(O)OAg)_*n*_ with a Lewis base (L), followed by I_2_ (in a 1:1:1 stoichiometry), gave iodine(I) pnictogenates of the form Ph_2_P(O)O─I─L (Fig. 2; Supplementary Note 3), which were achieved when L = 4-methylpyridine (4-Mepy; **1b**), 4-ethylpyridine (4-Etpy; **1c**), 4-dimethylaminopyridine (DMAP; **1d**), 4-pyrrolidinopyridine (4-pyrpy; **1e**), 4-piperidinopyridine (4-pippy; **1f**), 4-morpholinopyridine (4-morpy; **1g**). It should be noted that the IUPAC names would be: *pyridine iodine*(1+) *diphenyldioxidophosphate*(1−) (**1a**), and so forth, but will be shortened to iodine(I) phosphinates (for R_2_P(O)O derivatives), or more generally, to iodine(I) pnictogenates.

Interestingly, these phosphorus-based analogues differed in their apparent stabilities in solution, ranging from unstable (**1a**) to relatively stable in dry solvents (**1b**, **1c**, **1e**, **1g**), and even demonstrating apparent water-tolerance in non-treated solvents (**1d**, **1f**), which are important traits toward their appeal as prospective iodination reagents.

### Solution studies

The ^1^H NMR studies (Supplementary Figs. 1, 4, 7, 10, 13, 16) revealed predominantly only minor coordination shifts upon coordination of the free ligands (**b**–**g**) in comparison to their respective iodine(I) derivatives (**1b**–**1g**), with differences in the aromatic substituted pyridines ranging from 0.00 to 0.42 ppm, though most fell under 0.20 ppm, with only 4-morpy demonstrating notable coordination shifts of 0.26 and 0.42 ppm.

Prior observations of the carbonyl carbon atom (via ^13^C NMR studies) or of the fluorinated substituents of the carbonyl substituents (via ^19^F NMR studies) both found negligible coordination shifts upon iodine(I) formation^46,47^. However, the inclusion of a phosphorus atom, with its 100% abundance ^31^P nuclei *and* close proximity to the iodine atom, was envisioned to provide a valuable new NMR handle for these iodine(I) phosphinates. This proved true with notable, though modest, coordination shifts of 7.1–10.0 ppm in relation to (Ph_2_P(O)OAg)_*n*_ (18.4 ppm), though it should be noted that both these species were by necessity recorded in (CD_3_)_2_SO, preventing a direct comparison to the iodine(I) phosphinate complexes that were collected in CD_2_Cl_2_ (Supplementary Figs. 3, 6, 9, 12, 15, 18). Nevertheless, differences between the Lewis base incorporated in the iodine(I) phosphinates (all recorded in CD_2_Cl_2_) were also reflected in the ^31^P{^1^H} NMR chemical shifts (Supplementary Note 5), with values ranging from 25.5 ppm (**1e**) to 28.4 ppm (**1b**/**1c**).

Nevertheless, the solution studies were primarily focussed on the ^15^N NMR chemical shifts (determined from ^1^H–^15^N HMBC experiments; Supplementary Figs. 2, 5, 8, 11, 14, 17), as these have previously been demonstrated to show characteristic coordination shifts (Δδ_N_) of >100 ppm upon of the formation of iodine(I) complexes for both the [N─I─N]^+^ and C(O)O─I─N motifs^39,47,48^. The coordination shift is defined as the difference between the chemical shift of the coordinating (pyridinic) nitrogen atom in the free ligand (δ[^15^N_Ligand_]) and the respective iodine(I) complex (δ[^15^N_Complex_]) incorporating that same ligand and is usually reported as a positive value.

The free ligands (also in CD_2_Cl_2_) 4-Mepy (**b**, −75.1 ppm)^46^, 4-Etpy (**c**, −75.6 ppm)^49^, DMAP (**d**, −105.3 ppm)^13^, 4-pyrpy (**e**, −110.0 ppm)^50^, 4-pippy (**f**, −104.2 ppm)^39^, and 4-morpy (**g**, −99.1 ppm)^47^, were all found to demonstrate significant coordination shifts upon formation of the iodine(I) phosphinates (Supplementary Note 5), with Δδ_N_ values of 101.1 (**1b**; δ_N_ = −176.2), 99.6 (**1c**; δ_N_ = −175.2), 117.6 (**1d**; δ_N_ = −222.9), 118.1 (**1e**; δ_N_ = −228.1), 119.2 (**1f**; δ_N_ = −223.4), 116.3 (**1g**; δ_N_ = −215.4) ppm. These large Δδ_N_ values are consistent with prior observations for analogous iodine(I) carboxylate complexes^39,46,50^, though the iodine(I) pnictogenates demonstrated slightly larger values by comparison, e.g., PhC(O)O─I─(DMAP) (Δδ_N_ = 100.9) versus **1d** (Δδ_N_ = 117.6) and PhC(O)O─I─(4-pippy) (Δδ_N_ = 104.9) versus **1f** (Δδ_N_ = 119.2).

Unfortunately, complex **1a** could not be isolated in a pure form, as observed by the additional peaks in the ^1^H and ^31^P NMR spectra. Despite the ^15^N NMR chemical shift of −163.7 ppm observed in the reaction mixture giving a characteristic Δδ_N_ value of 96.0 ppm (cf. py (**a**) = −67.7 ppm)^13^, which is indicative of successful iodine(I) formation, the possibility of similar chemical shifts being observed for the protonated free ligands means these observations are ambiguous^15^.

### Solid-state studies

**Fig. 3:**
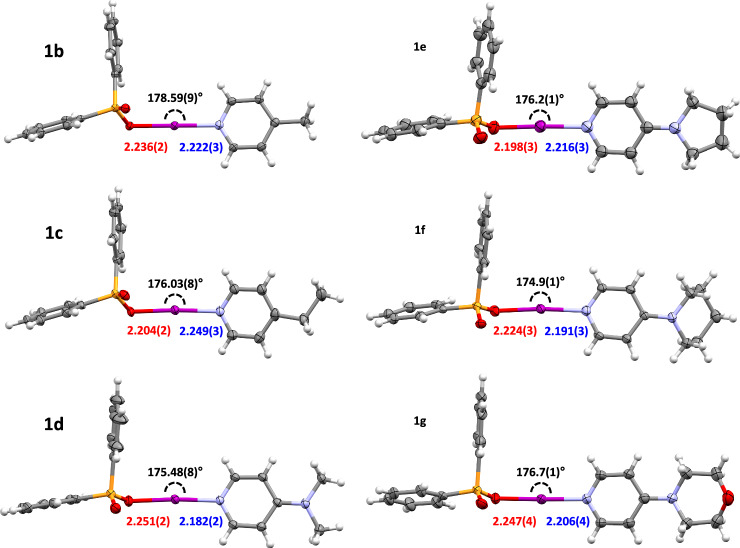
The solid-state structures of 1b–1g. Structures annotated with their O─I─N bond angles (black), as well as their O─I (red) and I─N (blue) bond lengths in Å (thermal ellipsoids at 50% probability; minor disordered positions and solvates omitted for clarity).

The solid-state structures for **1b**–**1g** were definitively established via single-crystal X-ray diffraction (SCXRD; Fig. 3), with the solid-state structure of **1a** understandably remaining elusive due to its high reactivity as observed in solution by NMR studies. In iodine(I) carboxylates, the relationship between the O─I and I─N bond lengths is such that strong Lewis bases generally cause shorter I─N (and concomitantly longer O─I) distances, and vice versa for weaker Lewis bases^39^, with retention of the overall intramolecular O···N distance that remains fairly constant (4.45 Å)^47,51^. These iodine(I) phosphinates also demonstrated fairly consistent intramolecular O···N distances for the halogen-bonded iodine atoms, despite the variation of their stabilising Lewis bases incorporated, with values ranging from 4.411(4) (**1b**) to 4.457(4) Å (**1e**/**1f**) (average = 4.43 Å). The I─N bond lengths did demonstrate the general trend of being shorter for the stronger Lewis bases, e.g., the shortest and longest I─N distances of 2.182(2) and 2.249(3) Å were for the DMAP (**1d**) and 4-Etpy (**1c**) derivatives, respectively. These inversely coincided with the range of O─I bond lengths of **1d** (2.251(2) Å) to **1e** (2.198(3) Å; *N.B*. the O─I distance for **1c** was 2.204(2) Å, which is crystallography indistinguishable to **1e** to a 3*σ* tolerance). The O─I─N angles were all effectively linear (180°), with an overall average of 176.3°, and the largest deviation from linearity observed of 5.1° (**1f**) being negligible. The formally inequivalent P=O and P─O bonds were always visibly differentiated in all the iodine(I) phosphinate derivatives, with narrow ranges of 1.481(3)–1.493(2) and 1.532(4)–1.546(2) Å, respectively, indicating distinct double- and single-bond character in their bonding.

In comparison to the closest matching iodine(I) carboxylate complex (Supplementary Note 7), PhC(O)O─I─L, for which only three solid-state structures of different Lewis bases (L) are known (L = py, DMAP, 4-morpy), the P(O)O─I─L complexes demonstrated generally shorter I─N (and longer O─I) bond lengths. This can be seen in the direct comparison of the O─I/I─N bond lengths of PhC(O)O─I─(DMAP) (2.210(3)/2.241(3) Å) with **1d** (2.251(2)/2.182(2) Å)^39^, and PhC(O)O─I─(4-morpy) (2.230(4)/2.232(4) Å) with **1g** (2.247(4)/2.206(4) Å)^39^, as well as the approximate comparison of PhC(O)O─I─(py) (O─I/I─N = 2.169(5)/2.292(6) and 2.159(5)/2.299(7) Å) with **1b** (2.236(2)/2.222(3) Å) and **1c** (2.204(2)/2.249(3) Å) for which no direct analogue is available for comparison^39,40^; in all these comparisons, the Ph_2_P(O)O─I derivatives are shorter beyond a 3*σ* tolerance when compared to the PhC(O)O─I derivatives. When compared to the only other known non-carbonyl O─I─L derivatives, that is, the sulfonate iodine(I) derivatives of the form RS(O)_2_O─I─L^44^, **1b**–**1g** possess longer I─N (and shorter O─I) bond lengths. This is illustrated by the direct comparison of the O─I/I─N bond lengths of MeS(O)_2_O─I─(4-Mepy) (2.347(4)/2.154(4) Å) and (tolyl)S(O)_2_O─I─(4-Mepy) (2.358(5)/2.142(4) Å) with **1b** (2.236(2)/2.222(3) Å), and of MeS(O)_2_O─I─(DMAP) (2.331(2)/2.140(3) Å) and (tolyl)S(O)_2_O─I─(DMAP) (2.339(2)/2.142(2) Å) with **1d** (2.251(2)/2.182(2) Å). These solid-state comparisons indicate that the iodine(I) pnictogenate complexes **1b**–**1g** lie in the penumbra between the less reactive carbonyl and unwieldy more reactive sulfonate O─I─N complexes, making them attractive prospects as potential iodination reagents.

**Fig. 4:**
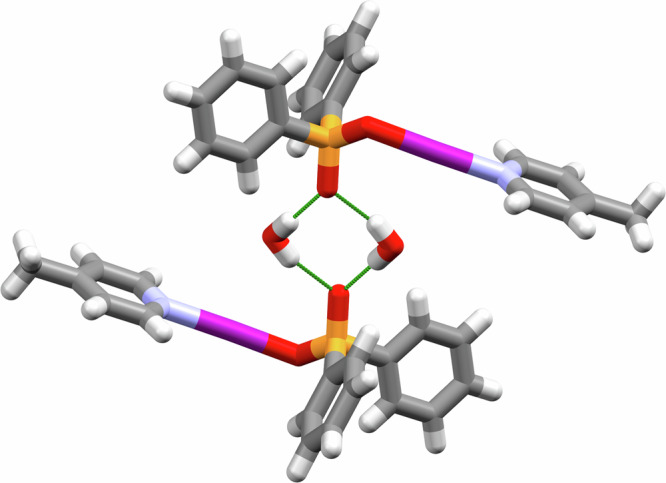
The packing of two molecules of 1b·H_2_O. The intermolecular hydrogen bonding motif of **1b** with two molecules of H_2_O (hydrogen bonds shown in green).

Interestingly, despite their aforementioned slight intolerance to water, only the monohydrated solid-state structures could be obtained for **1b**, **1c**, and **1g** (and also potentially **1d**, as an unknown solvate(s) in its solid-state structure had to be accounted for with Platon Squeeze) upon relaxation of the Schlenk handling techniques^52^. Attempts to obtain the non-hydrated solid-state structures for those complexes were unsuccessful, though such hydrated structures have been observed previously for even more unstable iodine(I) complexes^46^. For the solid-state structures of **1b**, **1c**, and **1g**, were all observed as 1:1 hydrates with a pair of H_2_O molecules bridging across two neighbouring P=O groups in a planar, diamond-shaped motif (Fig. 4). The possibility of the hydrogen-bonding inducing structural anomalies was considered, as had been observed previously in [N─I─N]^-^ complexes^53^, in comparison to the three non-hydrated solid-state structures **1d**, **1e**, and **1f**, though no structural anomalies were observed to support similar behaviour for the iodine(I) phosphinate complexes.

### Computational studies

**Fig. 5:**
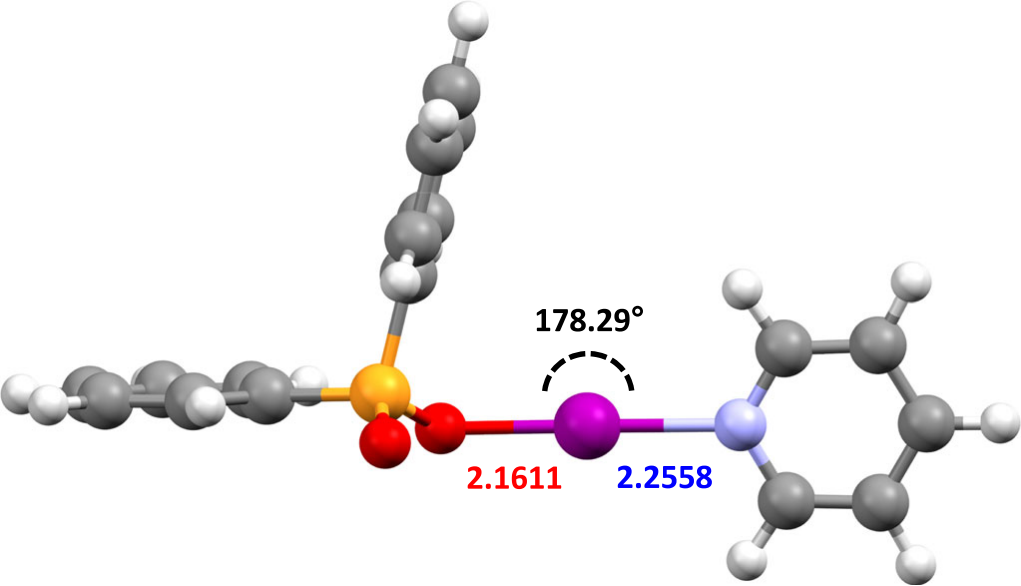
The calculated structure of 1a. Calculated using Spartan'20 at the M06-2X/def2-TZVP level of theory. Annotated with its O─I─N bond angle (black), as well as its O─I (red) and I─N (blue) bond lengths in Å.

The reliability of computational methods, which had been successfully utilised previously to excellently reproduce the molecular geometries obtained from SCXRD studies of [N─I─N]^+^ and RC(O)O─I─N (iodine(I) carboxylate) halogen-bonded iodine(I) complexes^13,15,23,39,46,49,54–57^, was also explored to see if they remained broadly applicable for this iodine(I) pnictogenate complexes (Supplementary Note 6). The geometry optimisations were performed at the M06-2X/def2-TZVP level of theory^58^ in the SPARTAN'20 programme^59^ with CH_2_Cl_2_ (dielectric = 8.82) as the solvent (Supplementary Notes 8, 9), and using a conductor-like polarisable continuum model (C-PCM)^60,61^. Additionally, this enabled the pyridine derivative **1a** (Fig. 5), for which the solid-state structure could not be obtained, to also be explored structurally.

In terms of the general geometric features, such as the intramolecular O···N distances, the computationally calculated models, **1a**_**calc**_–**1g**_**calc**_, showed excellent agreement with the SCXRD-determined structures, with an average O···N distance of 4.42 Å (cf. SCXRD = 4.43 Å). This was also observed for the Ph_2_P(O)O as a whole, with the averages of the calculated P─O and P=O bond lengths of 1.55 and 1.49 Å, respectively, matching well with the experimentally determined values (cf. 1.54 and 1.49 Å, respectively). Nevertheless, for the halogen-bonded bond lengths of interest, the accuracy was slightly diminished in the computational models, with differences ranging from 0.007–0.074 Å (O─I) and 0.004–0.036 Å (I─N). It should also be noted that in all but one case, the O─I bond lengths were underestimated, and the I─N bond lengths overestimated in the calculated models, with the one exception (**1e**) agreeing extremely well (O─I/I─N for **1e**: calc. = 2.2054/2.2119 Å; expt. = 2.198(3)/2.216(3) Å), such that these generalisations are not appropriate (to a 3*σ* tolerance, assuming approximate parity in e.s.d. values).

**Fig. 6:**
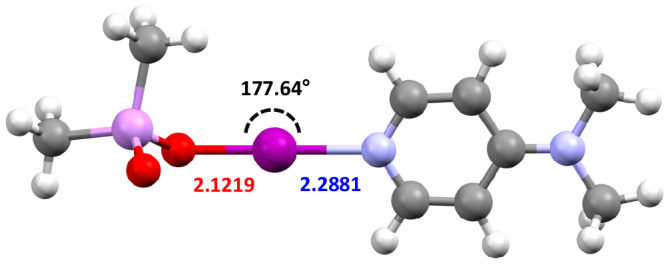
The calculated structure of Me_2_As(O)O─I─(DMAP). Calculated using Spartan'20 at the M06-2X/def2-TZVP level of theory. Annotated with its O─I─N bond angle (black), as well as its O─I (red) and I─N (blue) bond lengths in Å.

Whilst toxic, arsenic-based iodine(I) complexes would be pariahs as iodination reagents, especially toward the synthesis of pharmaceutical compounds, they were of interest to test the fundamental scope of iodine(I) complex synthesis on Group 15 elements. To this end, cacodylic acid (Me_2_As(O)OH) was also investigated in an analogous fashion to Ph_2_P(O)OH. Unfortunately, there was no literature procedure for the preparation of (Me_2_As(O)OAg)_*n*_, nor could this material be prepared in a similar fashion to the carboxylic and phosphinic acids without significant contamination, and was therefore ultimately abandoned. However, it should be noted that the corresponding As(O)O─I─N motif was proposed to be viable based on computational DFT studies (Fig. 6), which demonstrated comparable bond lengths/angles to known iodine(I) complexes.

### Reactivity studies

The reactivity of **1b**–**1g** as potential iodination reagents was also of particular interest, given that they represented a distinctly-different motif compared to prior O─I─N complexes primarily based on iodine(I) carboxylates (RC(O)O─I─L; L = Lewis base), in both the hybridisation and electronegativity of the oxygen-adjacent substituent (with *sp*^3^ P versus *sp*^2^ C). The utility of antipyrine’s straightforward conversion to iodo-antipyrine has been previously demonstrated^41,62,63^ and provides a useful benchmark in quantifying the reactivity of these iodine(I) phosphinates. The main limitation found for **1b**–**1g** was an inability to isolate and purify the bulk quantities necessary to proceed with such reactivity tests, with only **1d** and **1f** being found amenable. The importance of purifying the bulk material is due to the potential for the elemental iodine used in the synthesis of the iodine(I) pnictogenates also accomplishing the same iodination of antipyrine, and in *better* yield than observed under the same conditions for PhC(O)O─I─(DMAP)^41^.

These reactivity tests were performed over three reaction times (0.5, 2, and 22 h; Supplementary Note 4) in triplicate for **1d** and **1f**, which gave effectively indiscernible average percentage yields over the three reaction times tested (Table 1). When compared to [I(py)_2_]BF_4_ (Barluenga’s reagent), which can be considered the *industry standard* for such iodinations, [I(py)_2_]BF_4_ outperformed at 2 h with an average yield of 93%, though was identical at 0.5 h (65%) to **1d** (64%) and **1f** (65%). In comparison to I_2_, the iodine(I) phosphinates performed better at shorter reaction times (2 h) with yields of 75/73% (**1d**/**1f**) to the 55% observed for I_2_, though I_2_ gave higher yields (90%) at 22 h (cf. 77/76% for **1d**/**1f**). Most interestingly, though, was a marked improvement in comparison to the structurally similar iodine(I) carboxylates, with **1d** (75%) and **1f** (73%) at 2 h giving double the yields of PhC(O)O─I─(DMAP) (37%). Altogether, these comparisons show that the iodine(I) pnictogenates (**1d** and **1f**) can give respectable iodination yields that seem to crest at ~75–80% on a timescale analogous to currently utilised iodination reagents, and though they perform slightly worse than Barluenga’s reagent overall, they greatly outperform iodine(I) carboxylates.

**Table 1:** The average percentage conversions of antipyrine to iodo-antipyrine for complexes **1d** and **1f** over various reaction times, as well as other prior literature examples for comparison

Complex	0.5 h	2 h	22 h
**1d**	64	75	77
**1f**	65	73	76
[I(py)_2_]BF_4_^41,62^	65	93	–^a^
[I(DMAP)_2_]BF_4_^41^	–^b^	50	78
I_2_^41^	–^b^	55	90
PhC(O)OI(DMAP)^41^	–^b^	37	68

^a^The reactivity of [I(py)_2_]BF_4_ was not tested at 22 h due to its excellent yield after 2 h.

^b^The reactivity of [I(DMAP)2]BF_4_, I_2_, and PhC(O)OI(DMAP) was not tested at 0.5 h due to their underwhelming yields after 2 h.

**Fig. 7:**
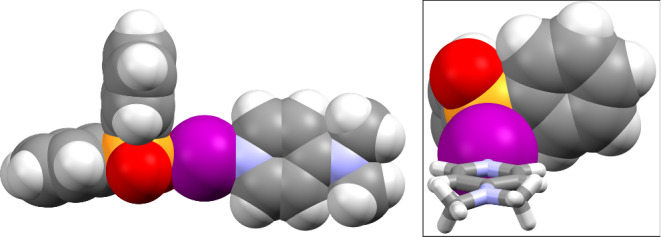
The spacefill representation of 1d. The orientation of the two phenyl substituents of **1d**, with one proximal to the iodine atom (inset: the same representation of **1d** viewed along the N─I─O bond with the DMAP ligand simplified for clarity).

**Fig. 8:**
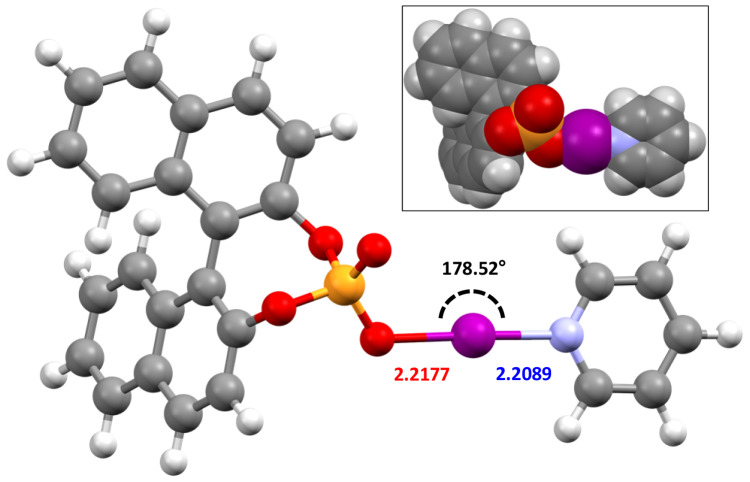
The calculated structure of (*S*-BINOL)P(O)O─I─(py). Calculated using Spartan'20 at the M06-2X/def2-TZVP level of theory. Annotated with its O─I─N bond angle (black), as well as its O─I (red) and I─N (blue) bond lengths in Å (inset: the spacefill representation of the same complex to better display the axial chirality of the *S*-BINOL substituent).

These surprising reactivity results support the potential of iodine(I) phosphinates as viable iodination reagents, which is a tantalising prospect if this can be combined with their other advantages. The structural studies of **1b**–**1g** had indicated that the tetrahedrally coordinated phosphorus atom of the Ph_2_P(O) substituent would project one of the Ph rings away from the iodine atom, whilst the other Ph ring would be proximal to the iodine atom (Fig. 7); a trait not possible in iodine(I) carboxylate complexes at the *sp*^2^-hybridised carbonyl carbon atom. In the iodine(I) pnictogenates this could potentially be utilised, most likely in analogous R_2_P(O) groups bearing bulkier R groups, to facially block one side of the iodine atom, which could be relevant toward future enantioselective applications. The ability to introduce chirality at the phosphorus atom, e.g., with enantiopure BINOL ([1,1’-binaphthalene]-2,2’-diol) derivatives of the form (BINOL)P(O)O─I─L (Fig. 8), much closer to the iodine atom than other prior examples^50,64^, is also a highly attractive prospect given that these proof-of-concept iodine(I) pnictogenate complexes demonstrate water tolerance in the solid state and respectable reactivity as iodination reagents.

## Conclusion

In conclusion, a series of iodine(I) pnictogenate complexes of the general form Ph_2_P(O)O─I─L (L = Lewis base) were synthesised via cation exchange of the respective silver(I) complex, (Ph_2_P(O)OAg)_*n*_, which itself was straightforwardly derived from Ph_2_P(O)OH. The procedure was analogously attempted for Me_2_As(O)OH, though the respective silver(I) precursor could not be prepared cleanly and was ultimately abandoned, though computational studies suggest that iodine(I) arsenate complexes of the form R_2_As(O)O─I─L would indeed be viable based on their calculated bond lengths and angles in comparison to known analogues. The synthesised iodine(I) phosphinates demonstrated decent stability and water tolerance in the solid state and were found to possess markedly improved reactivity as iodination reagents in comparison to their carbon-based analogues, iodine(I) carboxylates. Whilst not capable of yielding the same maximum yields as Barluenga’s reagent in terms of iodination when tested with antipyrine as the substrate, the iodine(I) phosphinates were found to be high-yielding within the same timeframe. The potential advantages of incorporating an *sp*^3^ phosphorus atom, compared to an *sp*^2^ carbon in iodine(I) carboxylates which have previously been the focus of recent studies, are a more responsive NMR-active nuclei (^31^P) and the ability to introduce chirality closer to the reactive iodine(I) centre. These advantages, in combination with their aforementioned excellent performance as iodination reagents, make iodine(I) pnictogenates an attractive new class of complexes with a bright future in the field of halogen bonding.

## Methods

### General considerations (Supplementary Note 1)

All reagents were obtained from commercial suppliers and used without further purification. Where required, reactions were carried out under an argon atmosphere using the Schlenk technique in oven-dried glassware, using a Schlenk line equipped with a gas‐drying unit. Dry CH_2_Cl_2_ was obtained by passing deoxygenated solvents through activated alumina columns (MBraun SPS‐800 Series solvent purification system) and stored under argon over 3 Å molecular sieves. The silver(I) precursor, (Ph_2_P(O)OAg)_*n*_, was prepared by an alternate procedure than previously described^45^. For structural NMR assignments, ^1^H NMR and ^1^H–^15^N NMR correlation spectra were recorded on a Bruker Avance III 500 MHz spectrometer in CD_2_Cl_2_. Chemical shifts are reported on the *δ* scale in ppm using the residual solvent signal as internal standard (CH_2_Cl_2_ in CD_2_Cl_2_: *δ*_H_ 5.32), or for ^1^H–^15^N NMR spectroscopy, to an external CD_3_NO_2_ standard. For the ^1^H NMR spectroscopy, each resonance was assigned according to the following conventions: chemical shift (*δ*) measured in ppm, observed multiplicity, observed coupling constant (*J* Hz), and number of hydrogens. Multiplicities are denoted as s (singlet), d (doublet), t (triplet), q (quartet), m (multiplet), and br (broad). For the ^1^H–^15^N HMBC spectroscopy, spectral windows of 4, 6, or 7 (6–10, 3–9, or 2–9; as required for nitrogen-based substituents) ppm (^1^H) and 300 ppm (^15^N) were used, with 1024 points in the direct dimension and 1024 increments used in the indirect dimension, with subsequent peak shape analysis being performed to give the reported ^15^N NMR resonances. The ^31^P{^1^H} NMR spectra were recorded on a Bruker Avance 300 MHz spectrometer, and chemical shifts are reported on the δ scale in ppm to an external H_3_PO_4_ standard. Multiplicities are denoted as s (singlet), d (doublet), t (triplet), q (quartet), m (multiplet), and br (broad).

### Single-crystal X-ray diffraction

The single crystal X-ray data were collected at 120 K using mirror-monochromated Cu-Kα (*λ* = 1.54184 Å) radiation on a Rigaku XtaLAB Synergy-R diffractometer with a HyPix-Arc 100 detector. All structures were solved by intrinsic phasing (SHELXT)^65^ and refined by full-matrix least-squares on *F*^2^ using Olex2^66^, utilising the SHELXL module^67^. Anisotropic displacement parameters were assigned to non-H atoms, and isotropic displacement parameters for all H atoms were constrained to multiples of the equivalent displacement parameters of their parent atoms with U_iso_(H) = 1.2 U_eq_(CH) or 1.5 U_eq_(CH_2_, CH_3_, OH) of their respective parent atoms. The SCXRD data for **1b**–**1g** (CCDC 2346048-2346053) are provided free of charge by the joint Cambridge Crystallographic Data Centre and Fachinformationszentrum Karlsruhe Access Structures service.

Please refer to the Supplementary Information for further details of the synthesis, characterisation and computational studies.

### Supplementary information

Supplementary Information

Description of Additional Supplementary Files

Supplementary Data 1

Supplementary Data 2

## Data Availability

All data generated or analysed during this study are included in this published article (and its supplementary information files). The X-ray crystallographic coordinates for all structures reported in this study (Supplementary Data 1) have been deposited at the Cambridge Crystallographic Data Centre (CCDC), under deposition numbers 2346048–2346053. These data can be obtained free of charge from The Cambridge Crystallographic Data Centre via www.ccdc.cam.ac.uk/data_request/cif. The combined Checkcif reports for all structures reported have also been generated and included (Supplementary Data 2).

## References

[1] Cavallo G (2016). The halogen bond. Chem. Rev..

[2] Gilday LC (2015). Halogen bonding in supramolecular chemistry. Chem. Rev..

[3] Pancholi J, Beer PD (2020). Halogen bonding motifs for anion recognition. Coord. Chem. Rev..

[4] Saccone M, Catalano L (2019). Halogen bonding beyond crystals in materials science. J. Phys. Chem. B.

[5] Desiraju GR (2013). Definition of the halogen bond (IUPAC Recommendations 2013). Pure Appl. Chem..

[6] Aakeröy CB (2012). The quest for a molecular capsule assembled via halogen bonds. CrystEngComm.

[7] Priimagi A (2012). Halogen bonding versus hydrogen bonding in driving self-assembly and performance of light-responsive supramolecular polymers. Adv. Funct. Mater..

[8] Dumele O, Trapp N, Diederich F (2015). Halogen bonding molecular capsules. Angew. Chem. Int. Ed..

[9] Szell PMJ, Zablotny S, Bryce DL (2019). Halogen bonding as a supramolecular dynamics catalyst. Nat. Commun..

[10] An S, Hao A, Xing P (2022). [N···I···N]^+^ type halogen-bonding-driven supramolecular helical polymers with modulated chirality. ACS Nano.

[11] An S, Hao A, Xing P (2023). Supramolecular axial chirality in [N–I–N]^+^-type halogen bonded dimers. Chem. Sci..

[12] Carlsson A-CC, Gräfenstein J, Laurila JL, Bergquist J, Erdélyi M (2012). Symmetry of [N–X–N]^+^ halogen bonds in solution. Chem. Commun..

[13] Ward JS, Fiorini G, Frontera A, Rissanen K (2020). Asymmetric [N–I–N]^+^ halonium complexes. Chem. Commun..

[14] Yu S, Ward JS (2022). Ligand exchange among iodine(I) complexes. Dalton Trans.

[15] Ward JS, Gomila RM, Frontera A, Rissanen K (2022). Iodine(I) complexes incorporating sterically bulky 2-substituted pyridines. RSC Adv..

[16] Ward JS (2022). The solid-state hierarchy and iodination potential of [bis(3-acetaminopyridine)iodine(I)]PF_6_. CrystEngComm.

[17] Ward JS, Sievänen EI, Rissanen K (2023). Solid-state NMR spectroscopy of Iodine(I) complexes.. Chem. Asian J..

[18] Kumar, P. et al. The impact of *ortho*-substituents on bonding in Silver(I) and Halogen(I) complexes of 2-mono- and 2,6-disubstituted pyridines: an in-depth experimental and theoretical study. *Chem. Eur. J.* **29**, e202303643 (2023). 10.1002/chem.202303643 38055221

[19] Turunen L (2016). [N⋅⋅⋅I^+^ ⋅⋅⋅N] Halogen-bonded dimeric capsules from Tetrakis(3-pyridyl)ethylene cavitands. Angew. Chem. Int. Ed..

[20] Turunen L, Warzok U, Schalley CA, Rissanen K (2017). Nano-sized I12L6 molecular capsules based on the [N⋅⋅⋅I^+^⋅⋅⋅N] halogen bond. Chem.

[21] Turunen L (2017). Tetrameric and dimeric [N⋅⋅⋅I^+^⋅⋅⋅N] halogen-bonded supramolecular cages. Chem. Eur. J..

[22] Taipale E (2022). Dimeric iodine(I) and silver(I) cages from tripodal N-donor ligands via the [N–Ag–N]^+^ to [N–I–N]^+^ cation exchange reaction. Inorg. Chem. Front..

[23] Yu S, Kalenius E, Frontera A, Rissanen K (2021). Macrocyclic complexes based on [N⋯I⋯N]^+^ halogen bonds. Chem. Commun..

[24] Hassel O, Hope H (1961). Structure of the solid compound formed by addition of two molecules of iodine to one molecule of pyridine. Acta Chem. Scand..

[25] Creighton, J. A., Haque, I. & Wood, J. L. The iododipyridinium ion. *Chem. Commun*. 229, (1966).

[26] Haque I, Wood JL (1968). The vibrational spectra and structure of the bis(pyridine)iodine(I), bis(pyridine)bromine(I), bis(γ-picoline)iodine-(I) and bis(γ-picollne)bromine(I) cations. J. Mol. Struct..

[27] Barluenga J, González JM, Campos PJ, Asensio GI (1985). (py)_2_BF_4_, a new reagent in organic synthesis: general method for the 1,2-Iodofunctionalization of olefins. Angew. Chem. Int. Ed..

[28] Barluenga, J., González, J. M., Garcia-Martin, M. A., Campos, P. J. & Asensio, G. An expeditious and general aromatic iodination procedure. *J. Chem. Soc. Chem. Commun*. 1016–1017 (1992).

[29] Ezquerra J (1996). Efficient reagents for the synthesis of 5-, 7-, and 5,7-substituted indoles starting from aromatic amines: scope and limitations. J. Org. Chem..

[30] Espuña, G. et al. Control of the iodination reaction on activated aromatic residues in peptides. *Chem. Commun*. 1307–1308 (2000).

[31] Barluenga J, González-Bobes F, Murguía MC, Ananthoju SR, González JM (2004). Bis(pyridine)iodonium tetrafluoroborate (IPy_2_BF_4_): a versatile oxidizing reagent. Chem. Eur. J..

[32] Tanner DD, Gidley GC (1968). Free-radical iodination. A novel synthetic method. J. Am. Chem. Soc..

[33] Barluenga J, González-Bobes F, González JM (2002). Activation of alkanes upon reaction with PhI(OAc)_2_–I_2_. Angew. Chem. Int. Ed..

[34] Montoro R, Wirth T (2003). Direct iodination of alkanes. Org. Lett..

[35] Kawasumi R (2017). One-step conversion of levulinic acid to succinic acid using I_2_/t-BuOK system: the iodoform reaction revisited. Sci. Rep..

[36] Tanner DD, Gidley GC, Das N, Rowe JE, Potter A (1984). On the structure of tert-butyl hypoiodite. J. Am. Chem. Soc..

[37] Courtneidge JL, Lusztyk J, Pagé D (1994). Alkoxyl radicals from alcohols. Spectroscopic detection of intermediate alkyl and acyl hypoiodites in the Suárez and Beebe reactions. Tetrahedron Lett..

[38] Hokamp T, Storm AT, Yusubov M, Wirth T (2018). Iodine monoacetate for efficient oxyiodinations of alkenes and alkynes. Synlett.

[39] Yu S, Ward JS, Truong K-N, Rissanen K (2021). Carbonyl hypoiodites as extremely strong halogen bond donors. Angew. Chem. Int. Ed..

[40] Hartl H, Hedrich M (1981). Crystal and molecular structures of benzoato-pyridine-iodine(I) and phthalato-bis(pyridine-iodine(I)). Z. Naturforsch. B.

[41] Wilson LME, Rissanen K, Ward JS (2023). Iodination of antipyrine with [N–I–N]^+^ and carbonyl hypoiodite iodine(I) complexes. New J. Chem..

[42] Ashikari Y, Shimizu A, Nokami T, Yoshida J (2013). Halogen and chalcogen cation pools stabilized by DMSO. Versatile reagents for alkene difunctionalization. J. Am. Chem. Soc..

[43] Muñiz K, García B, Martínez C, Piccinelli A (2017). Dioxoiodane compounds as versatile sources for Iodine(I) chemistry. Chem. Eur. J..

[44] Puttreddy, R., Kumar, P. & Rissanen, K. Pyridine Iodine(I) cations: kinetic trapping as a sulfonate complexes. *Chem. Eur. J.* **30**, e202304178 (2024). 10.1002/chem.202304178 38193788

[45] Moodley V, Mthethwa L, Pillay MN, Omondi B, van Zyl WE (2015). The silver(I) coordination polymer [AgO_2_PPh_2_]_*n*_ and unsupported Ag⋯Ag interactions derived from aminophosphinate and phosphinic acid. Polyhedron.

[46] Ward JS (2022). Carbonyl hypoiodites from pivalic and trimesic acid and their silver(I) intermediates. Dalton Trans..

[47] Kolařík, V., Rissanen, K. & Ward, J. S. Fluoro and trifluoromethyl benzoyl hypoiodite complexes with substituted pyridines. *Chem. Asian. J.* **19**, e202400349 (2024). 10.1002/asia.202400349 38578048

[48] Carlsson A-CC (2016). Substituent effects on the [N–I–N]^+^ halogen bond. J. Am. Chem. Soc..

[49] Kramer E, Yu S, Ward JS, Rissanen K (2021). Dihypoiodites stabilised by 4-ethylpyridine through O–I–N halogen bonds. Dalton Trans..

[50] Mattila M, Rissanen K, Ward JS (2023). Chiral carbonyl hypoiodites. Chem. Commun..

[51] Groom CR, Bruno IJ, Lightfoot MP, Ward SC (2016). The Cambridge structural database. Acta Crystallogr. Sect. B.

[52] Spek AL (2015). PLATON SQUEEZE: a tool for the calculation of the disordered solvent contribution to the calculated structure factors. Acta Crystallogr. Sect. C.

[53] Yu S (2023). Halogen-bonded [N–I–N]^−^ complexes with symmetric or asymmetric three-center–four-electron bonds. Cryst. Growth Des..

[54] Yu S, Kumar P, Ward JS, Frontera A, Rissanen KA (2021). A “nucleophilic” iodine in a halogen-bonded iodonium complex manifests an unprecedented I^+^···Ag^+^ interaction. Chem.

[55] Ward JS, Frontera A, Rissanen K (2021). Utility of three-coordinate silver complexes toward the formation of iodonium ions. Inorg. Chem..

[56] Kumar, P. et al. Linear bis-coordinate Silver(I) and Iodine(I) complexes with R_3_R_2_R_1_N tertiary amines. *Chem. Eur. J.* **29**, e202302162 (2023). 10.1002/chem.202302162 37682579

[57] Ward JS, Frontera A, Rissanen K (2021). Iodonium complexes of the tertiary amines quinuclidine and 1-ethylpiperidine. Dalton Trans..

[58] Zhao Y, Truhlar DG (2008). The M06 suite of density functionals for main group thermochemistry, thermochemical kinetics, noncovalent interactions, excited states, and transition elements: two new functionals and systematic testing of four M06-class functionals and 12 other function. Theor. Chem. Acc..

[59] Spartan’20 (Wavefunction Inc., Irvine, CA, USA, 2018).

[60] Zhang X, Herbert JM (2014). Excited-state deactivation pathways in uracil versus hydrated uracil: solvatochromatic shift in the 1nπ* state is the key. J. Phys. Chem. B.

[61] Lange AW, Herbert JM (2011). Symmetric versus asymmetric discretization of the integral equations in polarizable continuum solvation models. Chem. Phys. Lett..

[62] Campos PJ, Arranz J, Rodríguez MA (1997). α-Iodination of enaminones with bis(pyridine)iodonium(I) tetrafluoroborate. Tetrahedron Lett..

[63] Schumacher C (2024). Halogen bonding and mechanochemistry combined: synthesis, characterization, and application of N-iodosaccharin pyridine complexes. Org. Chem. Front..

[64] von der Heiden D (2021). Are bis(pyridine)iodine(I) complexes applicable for asymmetric halogenation?. Org. Biomol. Chem..

[65] Sheldrick GM (2015). SHELXT-integrated space-group and crystal-structure determination. Acta Crystallogr. Sect. A.

[66] Dolomanov OV, Bourhis LJ, Gildea RJ, Howard JAK, Puschmann H (2009). OLEX2: a complete structure solution, refinement and analysis program. J. Appl. Crystallogr..

[67] Sheldrick GM (2015). Crystal structure refinement with SHELXL. Acta Crystallogr. Sect. C.

