# Supplementary material for: Iodine(I) pnictogenate complexes as Iodination reagents
Source: Commun Chem. 2024 Jul 17;7:159. doi: 10.1038/s42004-024-01240-0 (PMC11255316; doi:10.1038/s42004-024-01240-0)
Supplement: Supplementary file 1 — Supplementary Information [file 42004_2024_1240_MOESM1_ESM.pdf]

Supplementary Information

Iodine(I) Pnictogenate Complexes as Iodination Reagents

Sharath Mohan[1], Kari Rissanen[1], and Jas S. Ward[1*]

[1] University of Jyvaskyla, Department of Chemistry, Jyväskylä 40014, Finland.

E-mail: james.s.ward@jyu.fi

Contents

Supplementary Methods S2

Supplementary Note 1: General Considerations S2

Supplementary Note 2: Silver(I) Precursor S3

Supplementary Note 3: Iodine(I) Pnictogenates S3

Supplementary Note 4: Reactivity Studies S6

Supplementary Note 5: Comparison Table of ^{31}P NMR and ^{15}N (δ_N/$\Delta\delta_N$) Chemical Shifts S6

Supplementary Note 6: SCXRD Versus Calculated (DFT) Comparison Table S7

Supplementary Note 7: Iodine(I) Pnictogenates Versus Iodine(I) Carboxylates SCXRD Comparison Table S8

NMR Spectra S9

Supplementary Computational Details S18

Supplementary Note 8: General Considerations S18

Supplementary Note 9: Cartesian Coordinates S19

Supplementary References S24

Supplementary Methods

Supplementary Note 1: General Considerations

All reagents were obtained from commercial suppliers and used without further purification. Where required, reactions were carried out under an argon atmosphere using Schlenk technique in oven-dried glassware, using a Schlenk line equipped with a gas-drying unit. Dry CH_2Cl_2 was obtained by passing deoxygenated solvents through activated alumina columns (MBraun SPS-800 Series solvent purification system), and stored under argon over 3 Å molecular sieves. The silver(I) precursor, $(Ph_2P(O)OAg)_n$, was prepared by an alternate procedure than previously described.[1] For structural NMR assignments, ^{1}H NMR and ^{1}H-^{15}N NMR correlation spectra were recorded on a Bruker Avance III 500 MHz spectrometer in CD_2Cl_2. Chemical shifts are reported on the δ scale in ppm using the residual solvent signal as internal standard (CH_2Cl_2 in CD_2Cl_2: δ_H 5.32), or for ^{1}H-^{15}N NMR spectroscopy, to an external CD_3NO_2 standard. For the ^{1}H NMR spectroscopy, each resonance was assigned according to the following conventions: chemical shift (δ) measured in ppm, observed multiplicity, observed coupling constant (*J* Hz), and number of hydrogens. Multiplicities are denoted as: s (singlet), d (doublet), t (triplet), q (quartet), m (multiplet), and br (broad). For the ^{1}H-^{15}N HMBC spectroscopy, spectral windows of 4, 6, or 7 (6-10, 3-9, or 2-9; as required for nitrogen-based substituents) ppm (^{1}H) and 300 ppm (^{15}N) were used, with 1024 points in the direct dimension and 1024 increments used in the indirect dimension, with subsequent peak shape analysis being performed to give the reported ^{15}N NMR resonances. The $^{31}P\{^{1}H\}$ NMR spectra were recorded on a Bruker Avance 300 MHz spectrometer, and chemical shifts are reported on the δ scale in ppm to an external H_3PO_4 standard. Multiplicities are denoted as: s (singlet), d (doublet), t (triplet), q (quartet), m (multiplet), and br (broad).

The single crystal X-ray data were collected at 120 K using mirror-monochromated Cu-Kα (λ = 1.54184 Å) radiation on a Rigaku XtaLAB Synergy-R diffractometer with a HyPix-Arc 100 detector. All structures were solved by intrinsic phasing (SHELXT)[2] and refined by full-matrix least squares on F^2 using Olex2,[3] utilising the SHELXL module.[4] Anisotropic displacement parameters were assigned to non-H atoms and isotropic displacement parameters for all H atoms were constrained to multiples of the equivalent displacement parameters of their parent atoms with $U_{iso}(H) = 1.2\ U_{eq}(CH)$ or $1.5\ U_{eq}(CH_2, CH_3, OH)$ of their respective parent atoms. The X-ray single crystal data and CCDC numbers (2346048-2346053) of all new structures are included below.

The following abbreviations are used: DMAP = 4-dimethylaminopyridine, 4-Etpy = 4-ethylpyridine, 4-Mepy = 4-methylpyridine (4-picoline), 4-morpy = 4-morpholinopyridine, py = pyridine, 4-pippy = 4-piperidinopyridine, 4-pyrpy = 4-pyrrolidinopyridine.

Supplementary Note 2: Silver(I) Precursor

$(Ph_2P(O)OAg)_n$: Diphenylphosphinic acid (0.545 g, 2.5 mmol) was suspended in H_2O (10 mL), followed by addition of a H_2O (3mL) solution of NaOH (0.1g, 2.5 mmol), and then stirred for 30 minutes to form a clear solution. A solution of $AgNO_3$ (0.425 g, 2.5 mmol) in H_2O (2 mL) was prepared. The sodium diphenylphosphinate intermediate was added drop wise to the $AgNO_3$ solution to give white precipitate. The white precipitate was isolated by filtration and washed in portions with H_2O (50 mL), EtOH (40 mL), Et_2O (30 mL), then further dried on the sinter to give an off-white solid. Yield = 0.664 g (82%).

Supplementary Note 3: Iodine(I) Pnictogenates

$Ph_2P(O)O$–I–(4-Mepy) (**1b**): The silver(I) complex, $(Ph_2P(O)OAg)_n$ (19.5 mg, 0.06 mmol), was suspended in DCM (4 mL) and to it was added pyridine (4.8 µL, 0.06 mmol), and then stirred for 5 minutes. I_2 (15.2 mg, 0.06 mmol) was added as a solid to the stirred solution to give a yellow precipitate and a peach-coloured solution, that was stirred for 10-15 minutes until all the iodine had been consumed and isolated by filtration. ^{1}H NMR (500 MHz, CD_2Cl_2): δ 8.45 (d, J = 5.8 Hz, 2H), 7.81 – 7.67 (m, 4H), 7.44 – 7.31 (m, 6H), 7.18 (d, J = 5.8 Hz, 2H), 2.42 (s, 3H); ^{1}H-^{15}N HMBC NMR (500 MHz, CD_2Cl_2): δ -176.3; ^{31}P NMR (122 MHz, CD_2Cl_2): δ 28.4. Crystals suitable for single X-ray diffraction analysis were obtained from a CH_2Cl_2 solution of **1b** vapour diffused with pentane at 253 K over seven days. Crystal data for **1b**: CCDC-2346048, $C_{18}H_{17}INO_2P{\cdot}H_2O$, M_r = 455.21, colourless plates, 0.12 × 0.07 × 0.01 mm, triclinic, space group P-1 (No. 2), a = 8.9987(2) Å, b = 9.4164(3) Å, c = 11.8309(5) Å, α = 72.528(3)°, β = 76.407(3)°, γ = 80.613(2)°, V = 924.82(5) $Å^3$, Z = 2, D_x = 1.635 Mg m^{-3}, F(000) = 452, µ = 14.55 mm^{-1}, T = 120 K, θ_{max} = 74.5°, 3779 independent reflections, 3464 reflections with I > 2σ(I), R_{int} = 0.047, 3779 reflections, 224 parameters, no restraints, GooF = 1.05, 0.57 < dΔρ < -0.87 e $Å^{-3}$, $R[F^2 > 2\sigma(F^2)]$ = 0.027, $wR(F^2)$ = 0.067.

$Ph_2P(O)O$–I–(4-Etpy) (**1c**): The silver(I) complex, $(Ph_2P(O)OAg)_n$ (19.5 mg, 0.06 mmol), was suspended in DCM (4 mL) and to it was added 4-Etpy (6.8 µL, 0.06 mmol), and then stirred for 5 minutes. I_2 (15.2 mg, 0.06 mmol) was added as a solid to the stirred solution to give a yellow precipitate and a pale-yellow solution, that was stirred for 10-15 minutes until all the iodine had been consumed and isolated by filtration. ^{1}H NMR (500 MHz, CD_2Cl_2) δ = 8.48 (d, J = 6.3 Hz, 2H), 7.74 (dd, J = 11.7 Hz, 7.5 Hz, 4H), 7.46 – 7.31 (m, 6H), 7.21 (d, J = 6.5 Hz, 2H), 2.72 (q, J = 7.6 Hz, 2H), 1.24 (t, J = 7.6 Hz, 3H); ^{1}H-^{15}N HMBC NMR (500 MHz, CD_2Cl_2): δ -175.2; ^{31}P NMR (122 MHz, CD_2Cl_2): δ 28.4. Crystals suitable for single X-ray diffraction analysis were obtained from a CH_2Cl_2 solution of **1c** vapour diffused with pentane at 253 K over seven days. Crystal data for **1c**: CCDC-2346049, $C_{19}H_{19}INO_2P{\cdot}H_2O$, M_r = 469.24, colourless plates, 0.11 × 0.05 × 0.02 mm, triclinic, space group P-1 (No. 2), a = 9.0653(2) Å, b = 9.2711(2) Å, c = 12.0375(2) Å, α = 75.857(1)°, β = 84.020(2)°, γ = 87.137(2)°, V = 975.38(3) $Å^3$, Z = 2, D_x = 1.598 Mg m^{-3}, F(000) = 468, µ = 13.82 mm^{-1}, T = 120 K, θ_{max} = 74.5°, 3983 independent reflections, 3667 reflections with I > 2σ(I), R_{int} = 0.066, 3983 reflections, 253 parameters, no restraints, GooF = 1.06, 1.06 < dΔρ < -0.69 e $Å^{-3}$, $R[F^2 > 2\sigma(F^2)]$ = 0.028, $wR(F^2)$ = 0.073.

$Ph_2P(O)O$–I–(DMAP) (**1d**): The silver(I) complex, $(Ph_2P(O)OAg)_n$ (19.5 mg, 0.06 mmol), was suspended in DCM (3 mL) and to it was added a DCM (1 mL) solution of DMAP (7.36 mg, 0.06 mmol), and then stirred for 5 minutes. I_2 (15.2 mg, 0.06 mmol) was added as a solid to the stirred solution to give a yellow precipitate and a pale-yellow solution, that was stirred for 10-15 minutes until all the iodine had been consumed and isolated by filtration. ^1H NMR (500 MHz, CD_2Cl_2): δ 8.03 (d, *J* = 6.7 Hz, 2H), 7.73 (td, *J* = 8.0 Hz, 3.8 Hz, 4H), 7.36 (qt, *J* = 8.2 Hz, 4.7 Hz, 6H), 6.37 (d, *J* = 7.2 Hz, 2H), 3.05 (s, 6H); ^1H-^{15}N HMBC NMR (500 MHz, CD_2Cl_2): δ -222.9 (pyridinic), -304.8 (NMe_2); ^{31}P NMR (122 MHz, CD_2Cl_2): δ 25.7. Crystals suitable for single X-ray diffraction analysis were obtained from a CH_2Cl_2 solution of **1d** vapour diffused with diisopropyl ether at ambient temperature over three days. Crystal data for **1d**: CCDC-2346050, $C_{19}H_{20}IN_2O_2P$, M_r = 466.24, colourless needles, 0.19 × 0.04 × 0.02 mm, triclinic, space group *P*-1 (No. 2), a = 7.3598(2) Å, b = 9.2439(2) Å, c = 15.5137(6) Å, α = 88.624(3)°, β = 78.608(3)°, γ = 75.919(2)°, V = 1003.24 (5) $Å^3$, Z = 2, D_x = 1.543 Mg m^{-3}, F(000) = 464, μ = 13.41 mm^{-1}, T = 120 K, $θ_{max}$ = 74.5°, 4088 independent reflections, 3813 reflections with I > 2σ(I), R_{int} = 0.047, 4088 reflections, 228 parameters, 36 restraints, GooF = 1.05, 0.65 < dΔρ < -0.76 e $Å^{-3}$, R[F^2 > 2σ(F^2)] = 0.026, *w*R(F^2) = 0.066.

$Ph_2P(O)O$–I–(4-pyrpy) (**1e**): The silver(I) complex, $(Ph_2P(O)OAg)_n$ (19.5 mg, 0.06 mmol), was suspended in DCM (3 mL) and to it was added a DCM (1 mL) solution of 4-pyrpy (8.89 mg, 0.06 mmol), and then stirred for 5 minutes. I_2 (15.2 mg, 0.06 mmol) was added as a solid to the stirred solution to give a yellow precipitate and a peach-coloured solution, that was stirred for 10-15 minutes until all the iodine had been consumed and isolated by filtration. ^1H NMR (500 MHz, CD_2Cl_2): δ 8.00 (d, *J* = 6.6 Hz, 2H), 7.80 – 7.68 (m, 4H), 7.43 – 7.29 (m, 6H), 6.25 (d, *J* = 6.6 Hz, 2H), 3.33 (s, 4H), 2.04 (s, 4H); ^1H-^{15}N HMBC NMR (500 MHz, CD_2Cl_2): δ -228.1 (the peak for the pyrrolidino nitrogen atom was not observed); ^{31}P NMR (122 MHz, CD_2Cl_2): δ 25.5. Crystals suitable for single X-ray diffraction analysis were obtained from a CH_2Cl_2 solution of **1e** vapour diffused with pentane at 253 K over seven days. Crystal data for **1e**: CCDC-2346051, $C_{21}H_{22}IN_2O_2P$, M_r = 492.27, yellow plate, 0.18 × 0.12 × 0.09 mm, monoclinic, space group *P*2$_1$/*n* , a = 11.4630(2) Å, b = 9.8435(1) Å, c = 18.8688(3) Å, β = 107.360(2)°, V = 2032.10(6) $Å^3$, Z = 4, D_x = 1.609 Mg m^{-3}, F(000) = 984, μ = 13.27 mm^{-1}, T = 120 K, $θ_{max}$ = 74.5°, 4155 independent reflections, 3995 reflections with I > 2σ(I), R_{int} = 0.032, 4155 reflections, 244 parameters, no restraints, GooF = 1.08, 0.73 < dΔρ < -0.86 e $Å^{-3}$, R[F^2 > 2σ(F^2)] = 0.035, *w*R(F^2) = 0.093.

$Ph_2P(O)O$–I–(4-pippy) (**1f**): The silver(I) complex, $(Ph_2P(O)OAg)_n$ (19.5 mg, 0.06 mmol), was suspended in DCM (3 mL) and to it was added a DCM (1 mL) solution of 4-pippy (9.73 mg, 0.06 mmol), and then stirred for 5 minutes. I_2 (15.2 mg, 0.06 mmol) was added as a solid to the stirred solution to give a yellow precipitate and a pale-yellow solution, that was stirred for 10-15 minutes until all the iodine had been consumed and isolated by filtration. ^1H NMR (500 MHz, CD_2Cl_2): δ 8.00 (d, *J* = 7.4 Hz, 2H), 7.84 – 7.65 (m, 4H), 7.36 (dd, *J* = 7.8 Hz, 2.9, 6H), 6.48 (d, *J* = 7.5 Hz, 2H), 3.42 (t, *J* = 5.7 Hz, 4H), 1.73 – 1.66 (m, 2H), 1.66 – 1.59 (m, 4H); ^1H-^{15}N HMBC NMR (500 MHz, CD_2Cl_2): δ -

223.5 (the peak for the piperidino nitrogen atom was not observed); ^{31}P NMR (122 MHz, CD_2Cl_2): δ 25.6. Crystals suitable for single X-ray diffraction analysis were obtained from a CH_2Cl_2 solution of **1f** vapour diffused with diisopropyl ether at ambient temperature over three days. Crystal data for **1f**: CCDC-2346052, $C_{22}H_{24}IN_2O_2P$, M_r = 506.30, colourless needles, 0.17 × 0.10 × 0.06 mm, monoclinic, space group $P2_1/n$, a = 9.1510(1) Å, b = 23.2007(3) Å, c = 10.0876(1) Å, β = 94.492(1)°, V = 2135.12(4) Å^3, Z = 4, D_x = 1.575 Mg m^{-3}, F(000) = 1016, μ = 12.65 mm^{-1}, T = 120 K, θ_{max} = 74.5°, 4372 independent reflections, 4095 reflections with I > 2σ(I), R_{int} = 0.050, 4375 reflections, 253 parameters, no restraints, GooF = 1.06, 0.76 < dΔρ < -0.87 e Å^{-3}, R[F^2 > 2σ(F^2)] = 0.035, wR(F^2) = 0.098.

$Ph_2P(O)O$–I–(4-morpy) (**1g**): The silver(I) complex, $(Ph_2P(O)OAg)_n$ (19.5 mg, 0.06 mmol), was suspended in DCM (3 mL) and to it was added a DCM (1 mL) solution of 4-morpy (9.85 mg, 0.06 mmol), and then stirred for 5 minutes. I_2 (15.2 mg, 0.06 mmol) was added as a solid to the stirred solution to give a yellow precipitate and a peach-coloured solution, that was stirred for 10-15 minutes until all the iodine had been consumed and isolated by filtration. ^{1}H NMR (500 MHz, CD_2Cl_2) δ 7.99 (d, J = 6.6 Hz, 2H), 7.78 – 7.69 (m, 4H), 7.42 – 7.29 (m, 6H), 6.25 (d, J = 6.6 Hz, 2H), 3.32 (s, 4H), 2.03 (s, 4H); ^{1}H-^{15}N HMBC NMR (500 MHz, CD_2Cl_2): δ -215.4 (pyridinic), -292.5 (morpholino); ^{31}P NMR (122 MHz, CD_2Cl_2): δ 26.4. Crystals suitable for single X-ray diffraction analysis were obtained from a CH_2Cl_2 solution of **1g** vapour diffused with pentane at 253 K over three days. Crystal data for **1g**: CCDC-2346053, $C_{21}H_{22}IN_2O_3P\cdot H_2O$, M_r = 526.29, colourless plate, 0.08 × 0.03 × 0.01 mm, monoclinic, space group $P2_1/c$, a = 12.4883(4) Å, b = 18.8866(4) Å, c = 9.2728(3) Å, β = 98.864(3)°, V = 2160.98(11) Å^3, Z = 4, D_x = 1.618 Mg m^{-3}, F(000) = 1056, μ = 12.60 mm^{-1}, T = 120 K, θ_{max} = 74.5°, 4421 independent reflections, 3442 reflections with I > 2σ(I), R_{int} = 0.059, 4421 reflections, 268 parameters, no restraints, GooF = 1.05, 1.22 < dΔρ < -1.17 e Å^{-3}, R[F^2 > 2σ(F^2)] = 0.042, wR(F^2) = 0.106.

Supplementary Note 4: Reactivity Studies

The general procedure for testing the reactivity of the iodine(I) complexes, as outlined in previous research,[5–7] was conducted in triplicate for greater accuracy and involved the following steps:

Antipyrine (18.8 mg, 0.1 mmol) was added to a CH_2Cl_2 (5 mL) solution of the iodine(I) complex (0.1 mmol) being tested and stirred for the specified amount of time (0.5, 2, or 22 hours), followed by an aqueous work-up consisting of washing the reaction mixture with a saturated $NaHCO_3$ solution (4 × 25 mL). The iodo-antipyrine product was extracted with CH_2Cl_2, dried over anhydrous Na_2SO_4 and filtered, then isolated under reduced pressure as a white solid, from which the percentage yield was calculated and the purity confirmed via ^{1}H NMR spectroscopy.

Supplementary Table 1: The average percentage conversions (with individual values given in brackets) of antipyrine to iodo-antipyrine for complexes **1d** and **1f** over various reaction times, as well as other prior literature examples for comparison.

Complex	0.5 hours	2 hours	22 hours
**1d**	64% (61/64/66)	75% (74/75/75)	77% (77/77/78)
**1f**	65% (63/65/66)	73% (73/73/73)	76% (76/76/77)
**$[I(py)_2]BF_4$** (*Barluenga's reagent*)	65% (64/65/66)	93%[6]	-
**$[I(DMAP)_2]BF_4$**	-	50%[6]	78%[6]
**I_2**	-	55%[6]	90%[6]
**PhC(O)O–I–(DMAP)**	-	37%[6]	68%[6]

Supplementary Note 5: Comparison Table of ^{31}P NMR and ^{15}N ($\delta_N/\Delta\delta_N$) Chemical Shifts

Supplementary Table 2: The ^{15}N (pyridinic nitrogen) and ^{31}P NMR chemical shifts (in CD_2Cl_2), as well as the coordination shifts ($\Delta\delta_N$) for the iodine(I) pnictogenate complexes reported herein.

Complex	δ ^{31}P	δ ^{15}N (pyridinic)	$\Delta\delta_N$
**1b**	28.4	-176.2	101.1
**1c**	28.4	-175.2	99.6
**1d**	25.7	-222.9	117.6
**1e**	25.5	-228.1	118.1
**1f**	25.6	-223.4	119.2
**1g**	26.4	-215.4	116.3

Free ligands (in CD_2Cl_2): 4-Mepy (**b**, -75.1 ppm),[8] 4-Etpy (**c**, -75.6 ppm),[9] DMAP (**d**, -105.3 ppm),[10] 4-pyrpy (**e**, -110.0 ppm),[11] 4-pippy (**f**, -104.2 ppm),[12] and 4-morpy (**g**, -99.1 ppm).[8]

Supplementary Note 6: SCXRD Versus Calculated (DFT) Comparison Table

Supplementary Table 3: The O–I and I–N (in Å) bond lengths, and the O–I–N bond angles (in °) for complexes **1b**-**1g** and $Me_2As(O)O$–I–(DMAP) (**2d**) as determined by SCXRD and from computationally-calculated theoretical models.

**Complex**	**O–I (Å)** [SCXRD]	**O–I (Å)** [Calc.]	**I–N (Å)** [SCXRD]	**I–N (Å)** [Calc.]	**O–I–N (°)** [SCXRD]	**O–I–N (°)** [Calc.]	**O···N (Å)** [SCXRD]	**O···N (Å)** [Calc.]
**1a**†	-	2.1611	-	2.2558	-	178.29	-	4.4165
**1b**	2.236(2)	2.1616	2.222(3)	2.2581	178.59(9)	178.21	4.457(4)	4.4193
**1c**	2.204(2)	2.1637	2.249(3)	2.2552	176.03(8)	177.84	4.450(4)	4.4182
**1d**	2.251(2)	2.2037	2.182(2)	2.2143	175.48(8)	178.42	4.429(3)	4.4175
**1e**	2.198(3)	2.2054	2.216(3)	2.2119	176.2(1)	178.32	4.411(4)	4.4169
**1f**	2.224(3)	2.2006	2.191(3)	2.2168	174.9(1)	178.50	4.411(4)	4.4170
**1g**	2.247(4)	2.1903	2.206(4)	2.2271	176.7(1)	178.11	4.451(6)	4.4168
**2d**	-	2.1219	-	2.2881	-	177.64	-	4.4091
**(*S*-BINOL) P(O)-I-(py)**	-	2.2177	-	2.2089	-	178.52	-	4.4261

† The solid-state structure of **1a** was not isolated.

Supplementary Note 7: Iodine(I) Pnictogenates Versus Iodine(I) Carboxylates SCXRD Comparison Table

Supplementary Table 4: The SCXRD comparisons of the O–I and I–N bond lengths (in Å) of iodine(I) pnictogenates and their closest iodine(I) complex analogues.

**Complex**	**O–I (Å)**	**I–N (Å)**	**Complex**	**O–I (Å)**	**I–N (Å)**	**Complex**	**O–I (Å)**	**I–N (Å)**
**1b**	2.236(2)	2.222(3)	**1d**	2.251(2)	2.182(2)	**1g**	2.247(4)	2.206(4)
PhC(O)O–I–(py)[12]*	2.169(5)<br>2.159(5)	2.292(6)<br>2.299(7)	PhC(O)O–I–(DMAP)[12]	2.251(2)	2.182(2)	PhC(O)O–I–(4-morpy)[12]	2.247(4)	2.206(4)
MeS(O)$_2$O–I–(4-Mepy)[13]	2.347(4)	2.154(4)	MeS(O)$_2$O–I–(DMAP)[13]	2.331(2)	2.140(3)			
(tolyl)S(O)$_2$O–I–(4-Mepy)[13]	2.358(5)	2.142(4)	(tolyl)S(O)$_2$O–I–(DMAP)[13]	2.339(2)	2.142(2)			

***** The solid-state structure of the direct analogue PhC(O)O–I–(4-Mepy) has not been previously reported.

NMR Spectra

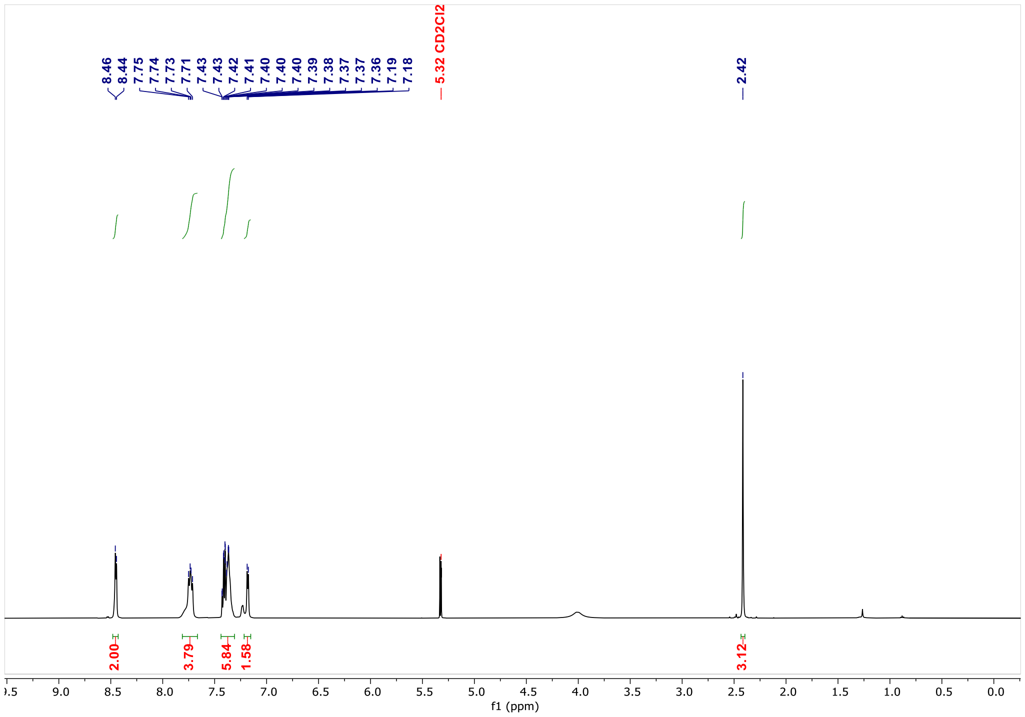

Supplementary Figure 1: The ^{1}H NMR spectrum of **1b** (CD_2Cl_2, 500 MHz, 298 K).

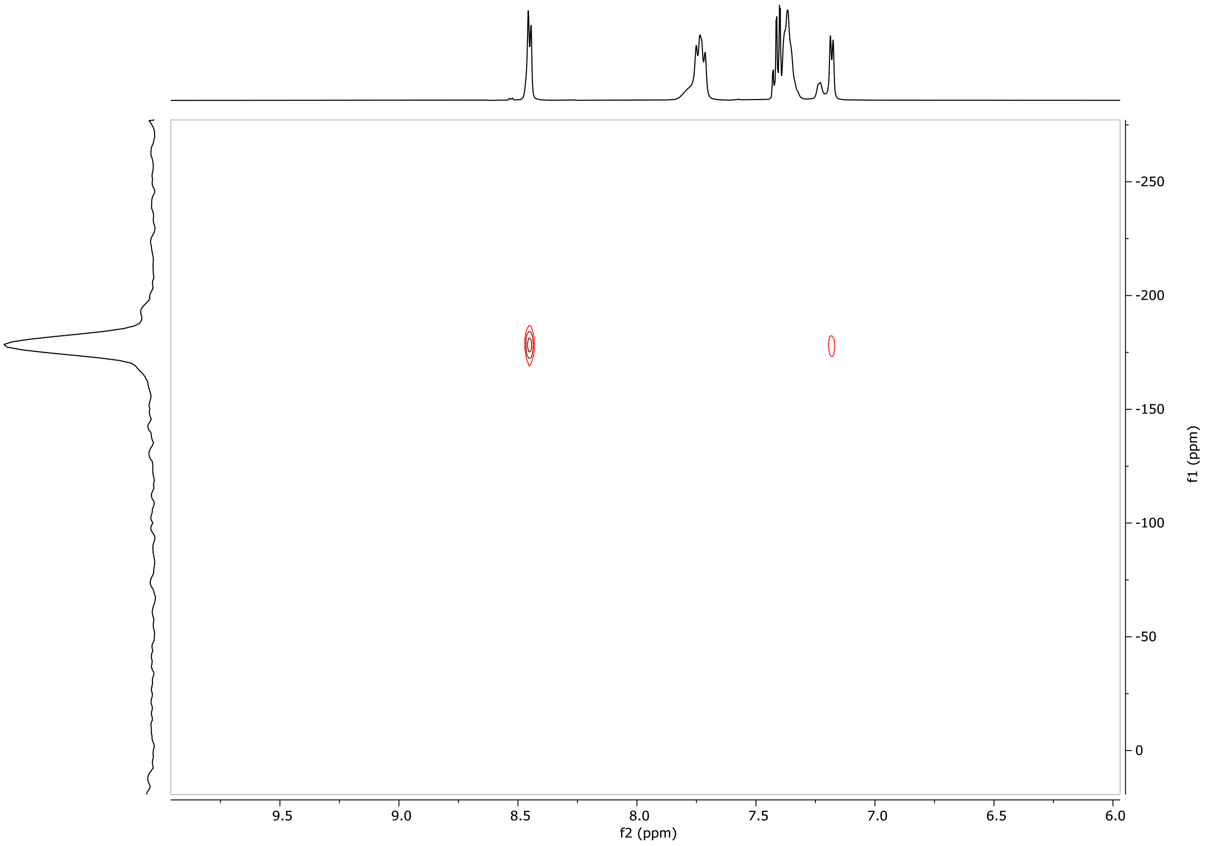

Supplementary Figure 2: The ^{1}H-^{15}N HMBC spectrum of **1b** (CD_2Cl_2, 298 K).

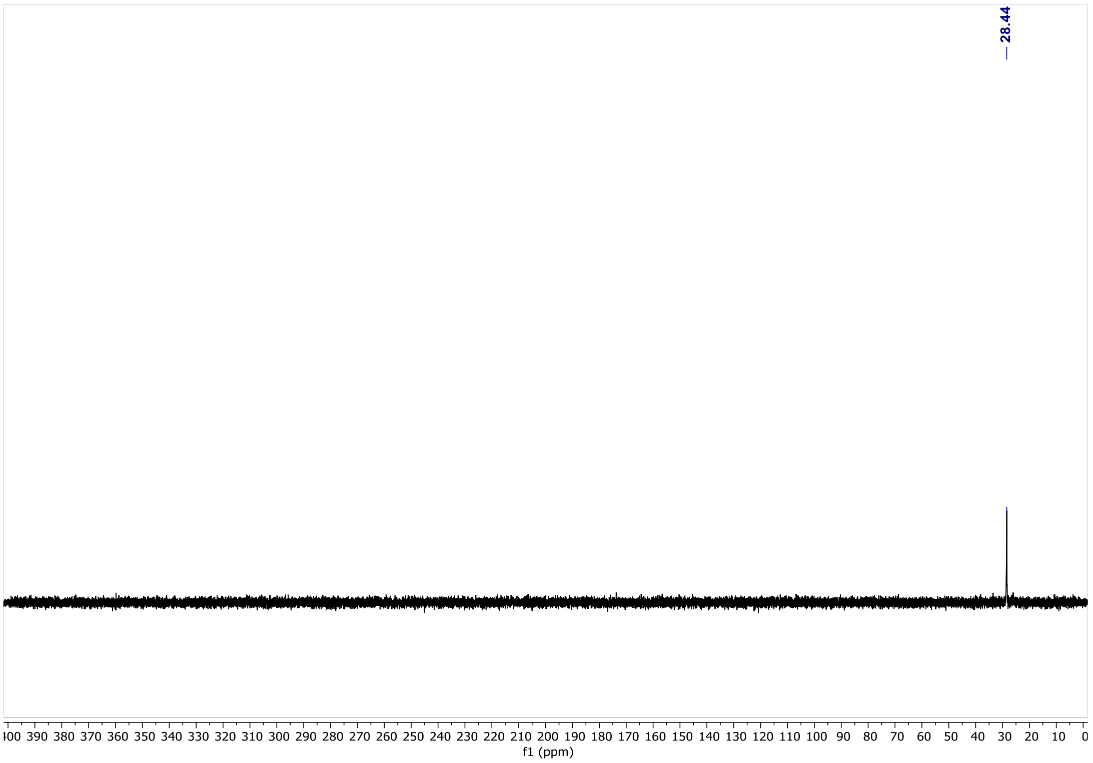

Supplementary Figure 3: The ^{31}P NMR spectrum of **1b** (CD_2Cl_2, 122 MHz, 298 K).

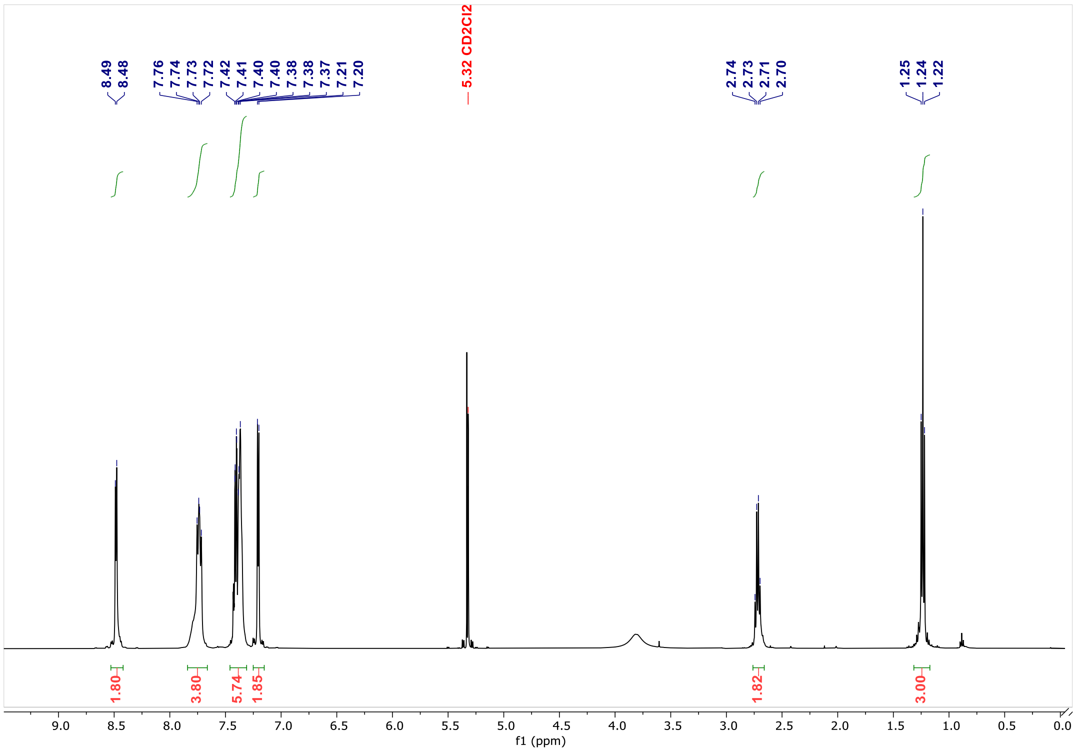

Supplementary Figure 4: The ^{1}H NMR spectrum of **1c** (CD_2Cl_2, 500 MHz, 298 K).

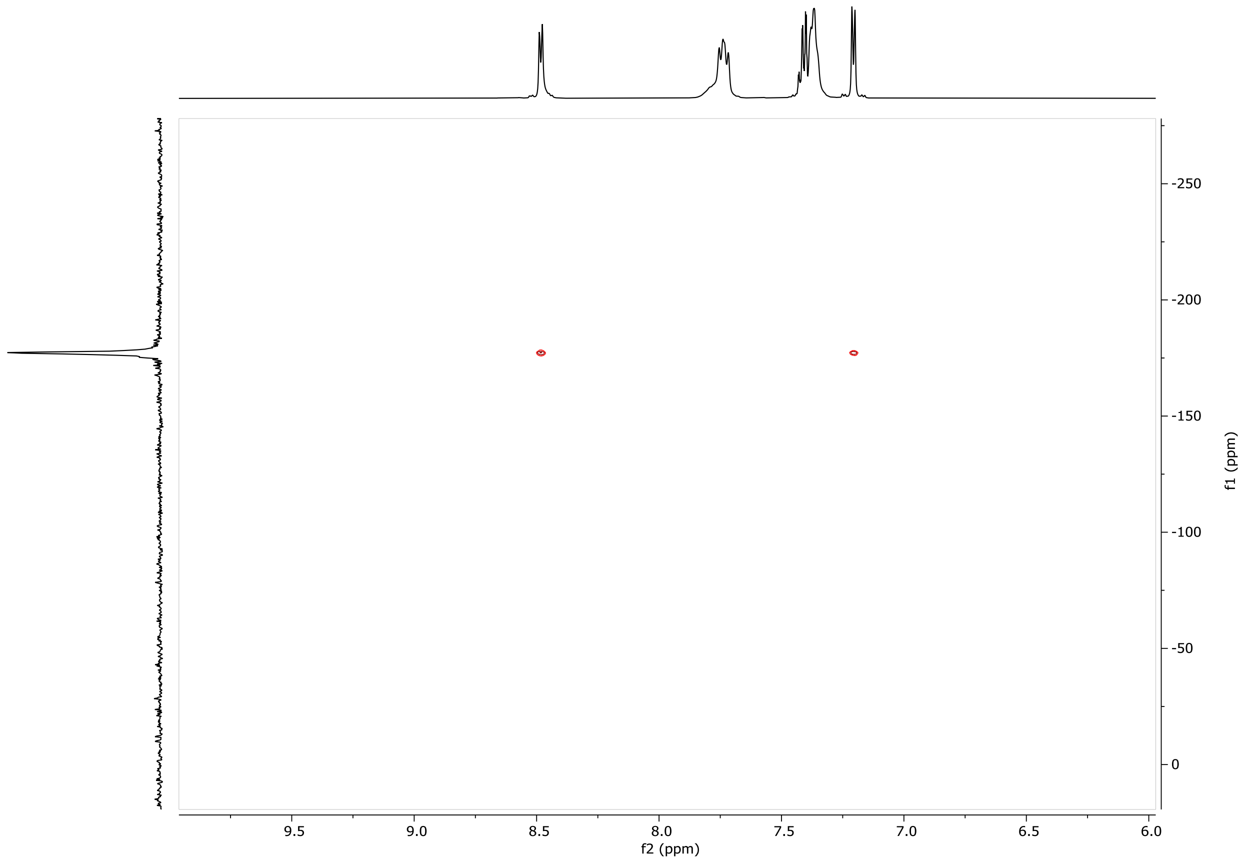

Supplementary Figure 5: The ^{1}H-^{15}N HMBC spectrum of **1c** (CD_2Cl_2, 298 K).

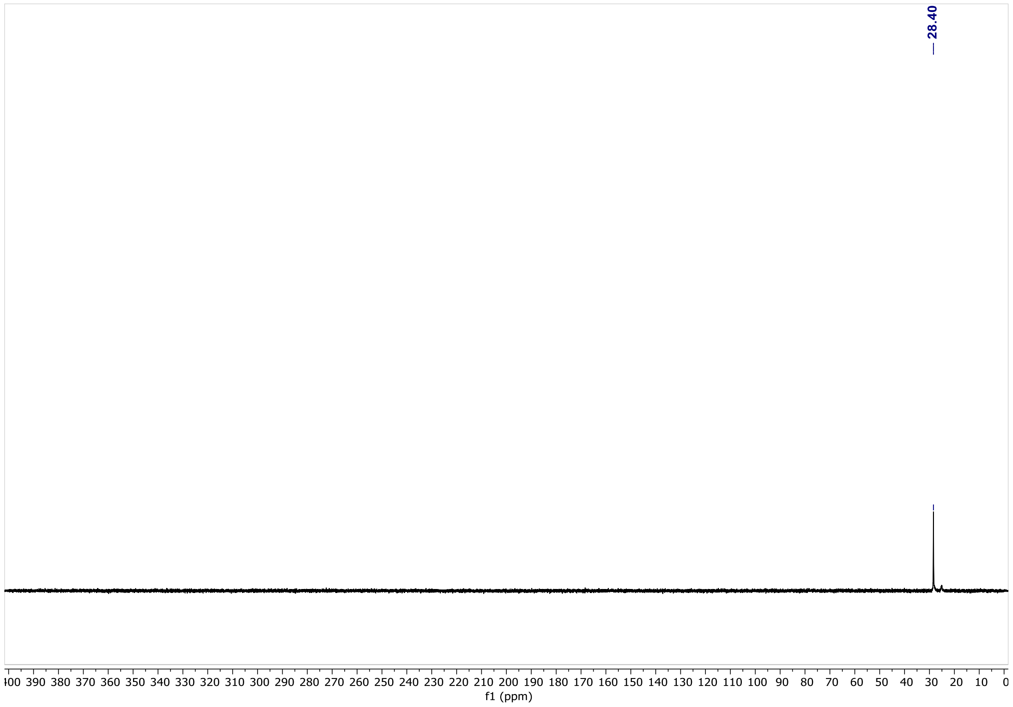

Supplementary Figure 6: The ^{31}P NMR spectrum of **1c** (CD_2Cl_2, 122 MHz, 298 K).

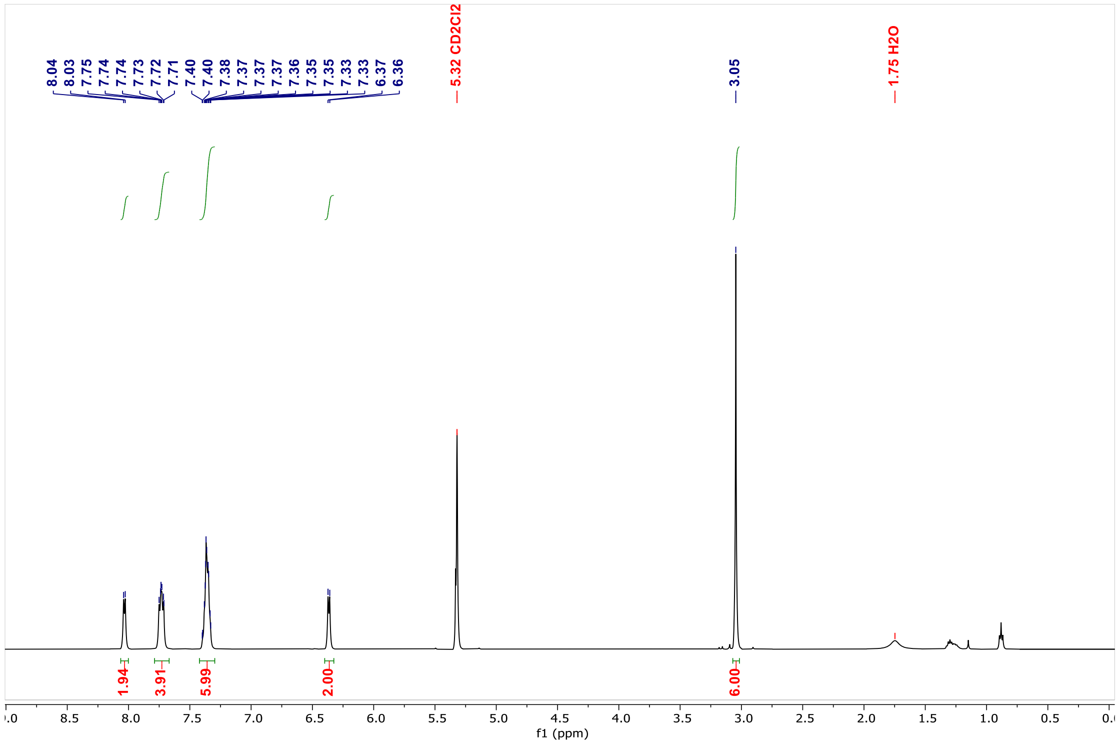

Supplementary Figure 7: The ^{1}H NMR spectrum of **1d** (CD_2Cl_2, 500 MHz, 298 K).

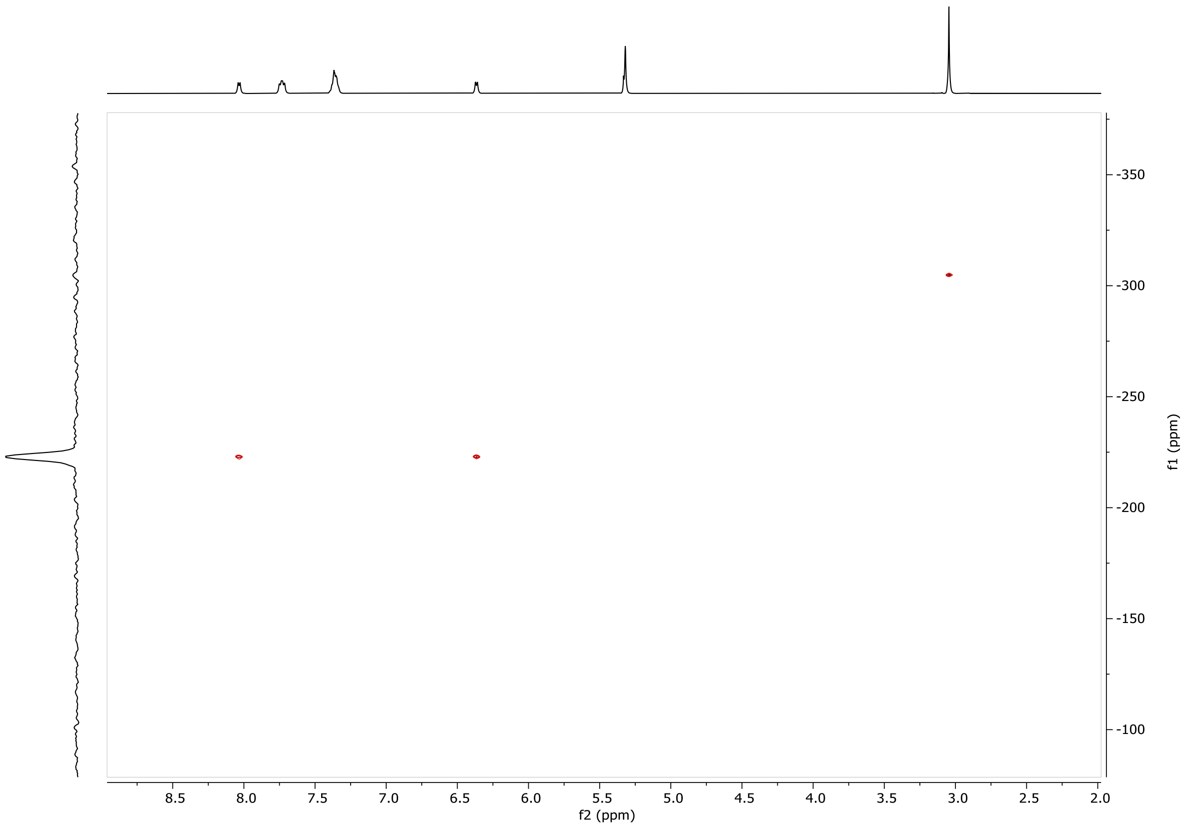

Supplementary Figure 8: The ^{1}H-^{15}N HMBC spectrum of **1d** (CD_2Cl_2, 298 K).

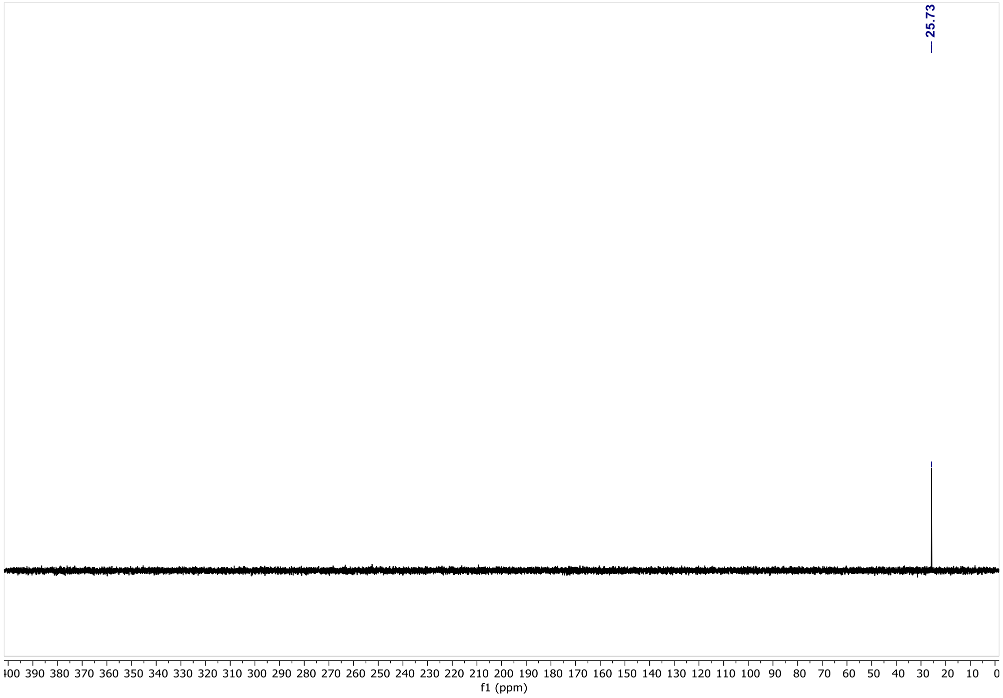

Supplementary Figure 9: The ^{31}P NMR spectrum of **1d** (CD_2Cl_2, 122 MHz, 298 K).

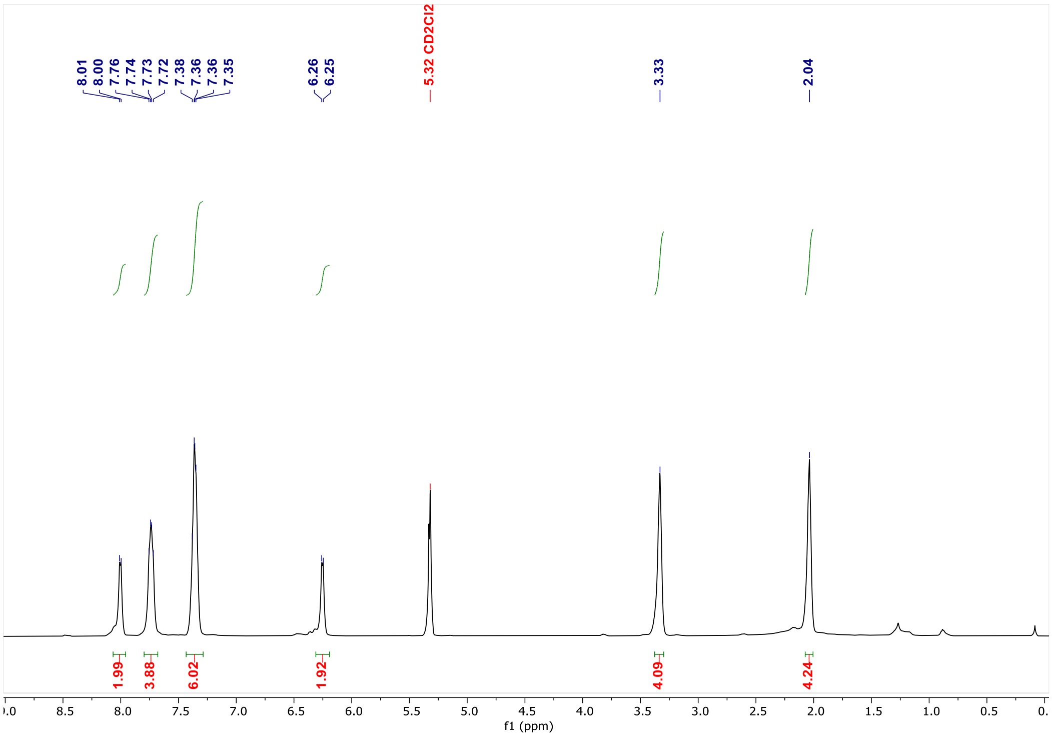

Supplementary Figure 10: The ^{1}H NMR spectrum of **1e** (CD_2Cl_2, 500 MHz, 298 K).

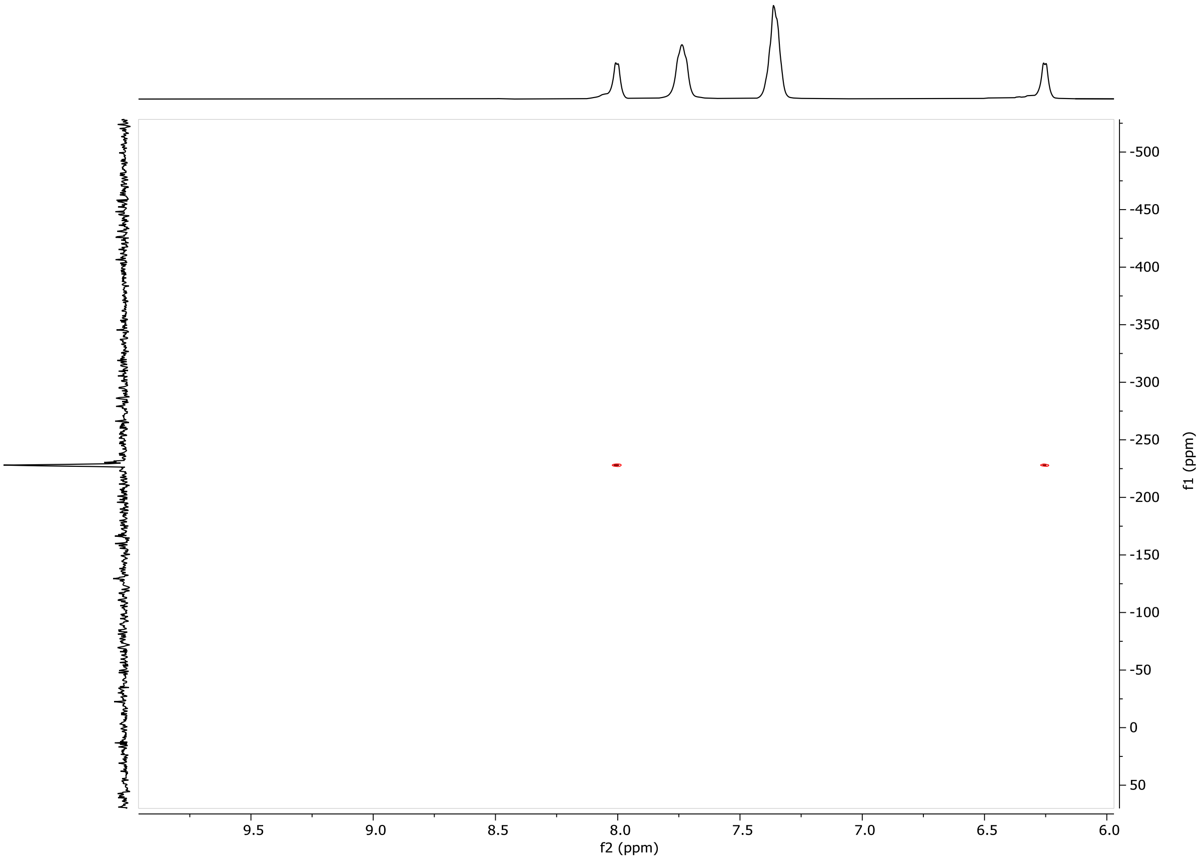

Supplementary Figure 11: The ^{1}H-^{15}N HMBC spectrum of **1e** (CD_2Cl_2, 298 K).

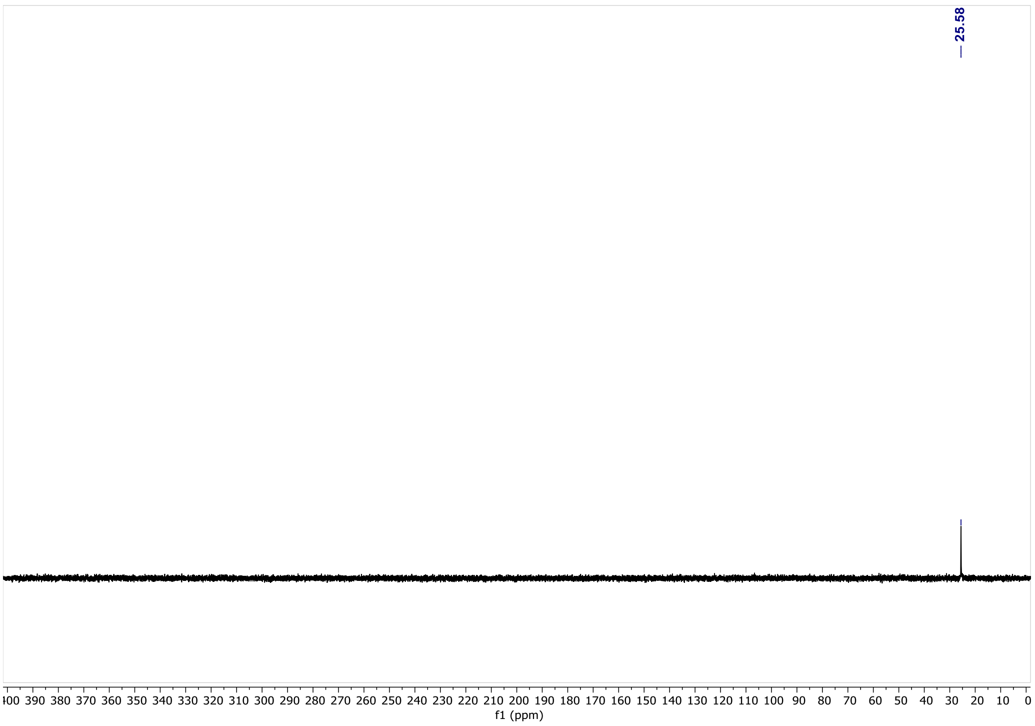

Supplementary Figure 12: The ^{31}P NMR spectrum of **1e** (CD_2Cl_2, 122 MHz, 298 K).

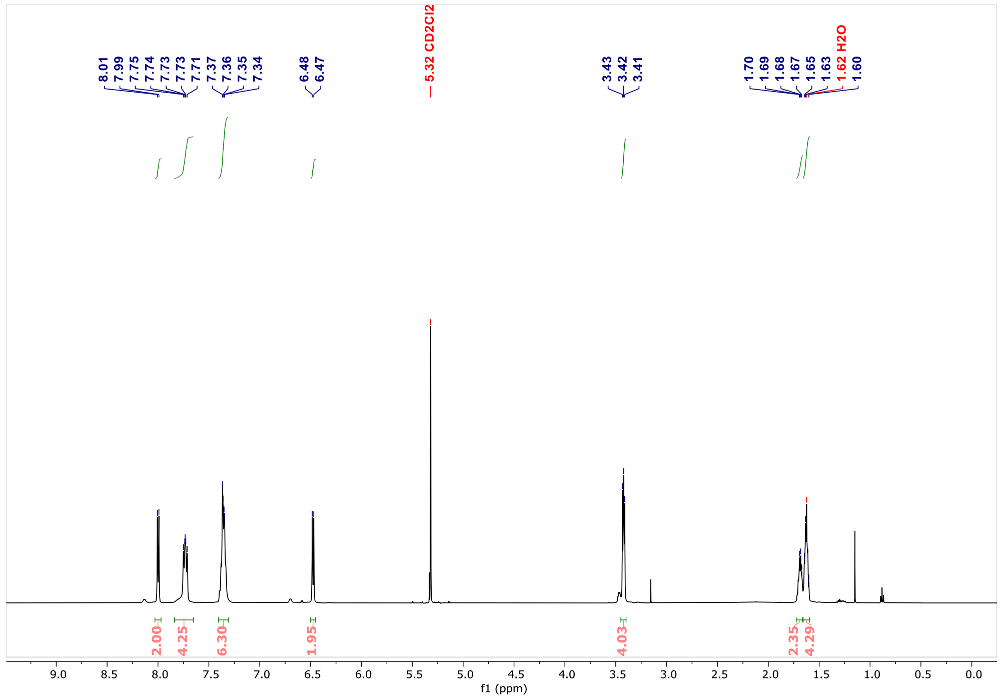

Supplementary Figure 13: The ^{1}H NMR spectrum of **1f** (CD_2Cl_2, 500 MHz, 298 K).

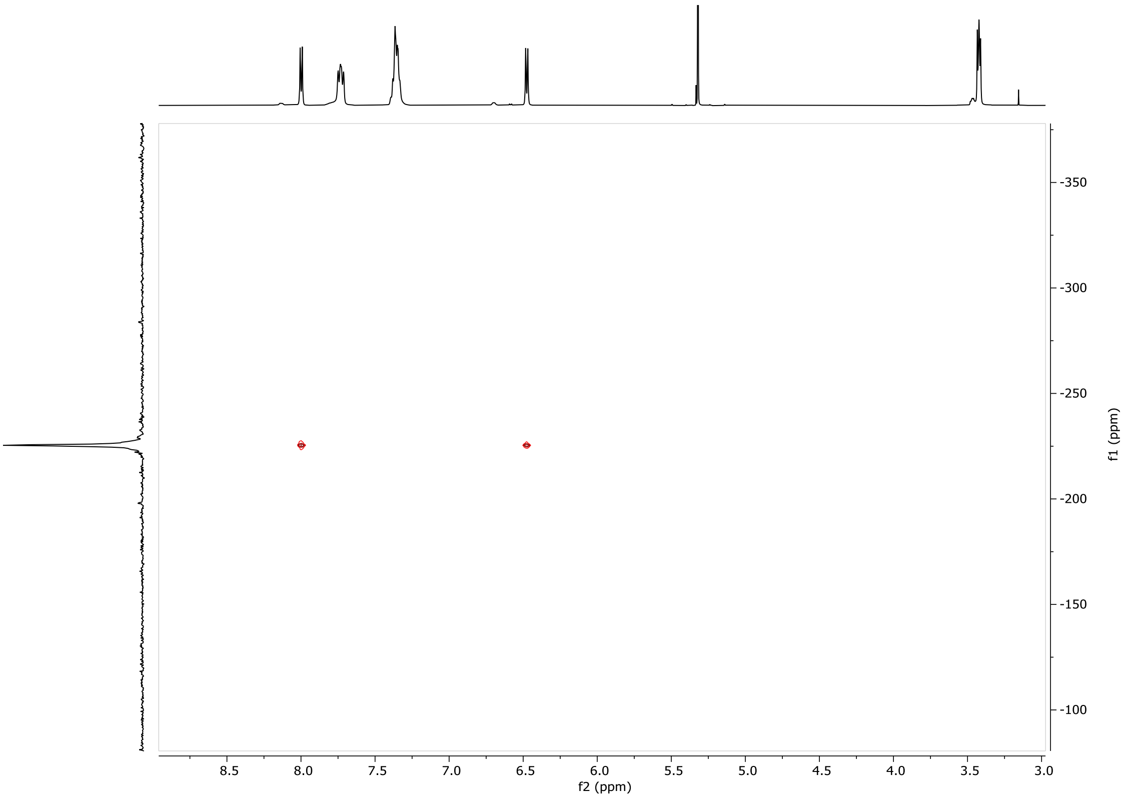

Supplementary Figure 14: The ^{1}H-^{15}N HMBC spectrum of **1f** (CD_2Cl_2, 298 K).

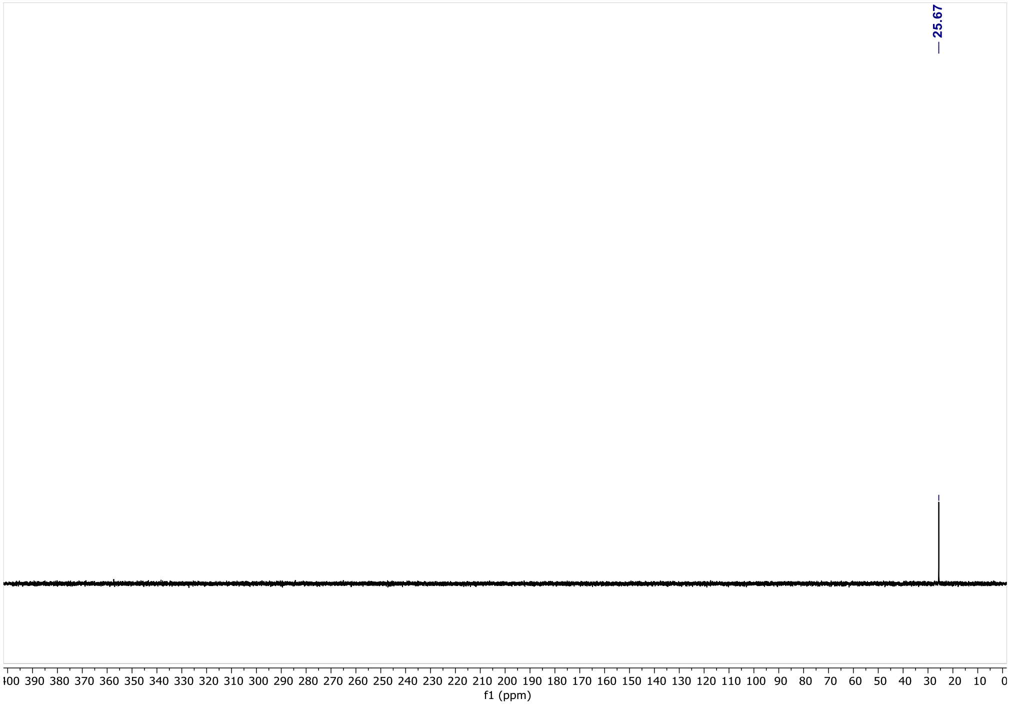

Supplementary Figure 15: The ^{31}P NMR spectrum of **1f** (CD_2Cl_2, 122 MHz, 298 K).

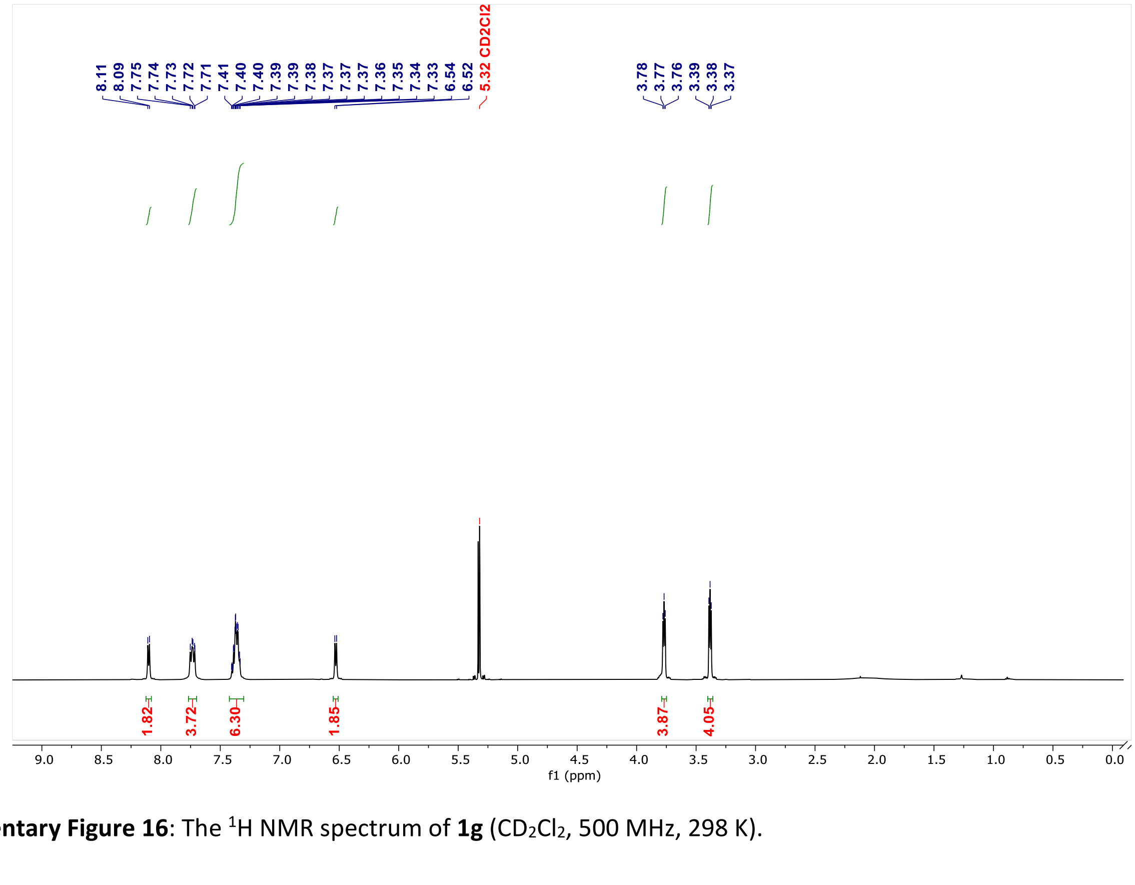

Supplementary Figure 16: The ^{1}H NMR spectrum of **1g** (CD_2Cl_2, 500 MHz, 298 K).

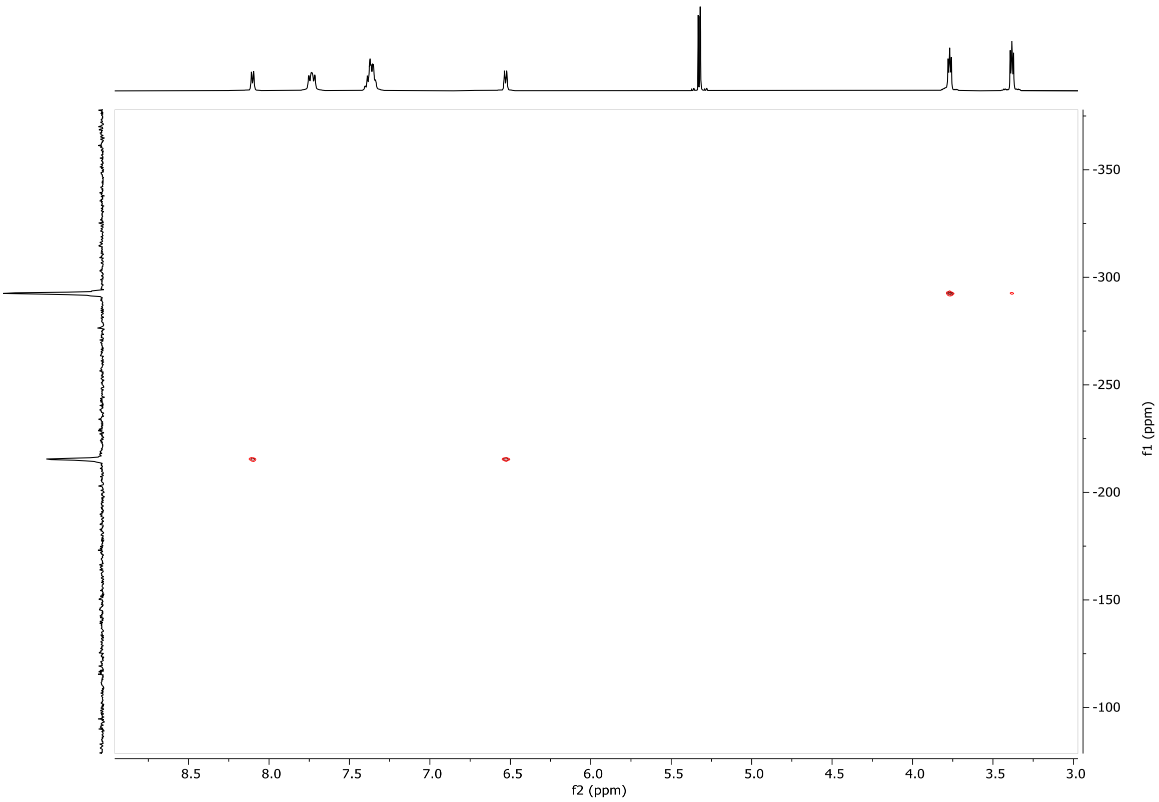

Supplementary Figure 17: The ^{1}H-^{15}N HMBC spectrum of **1g** (CD_2Cl_2, 298 K).

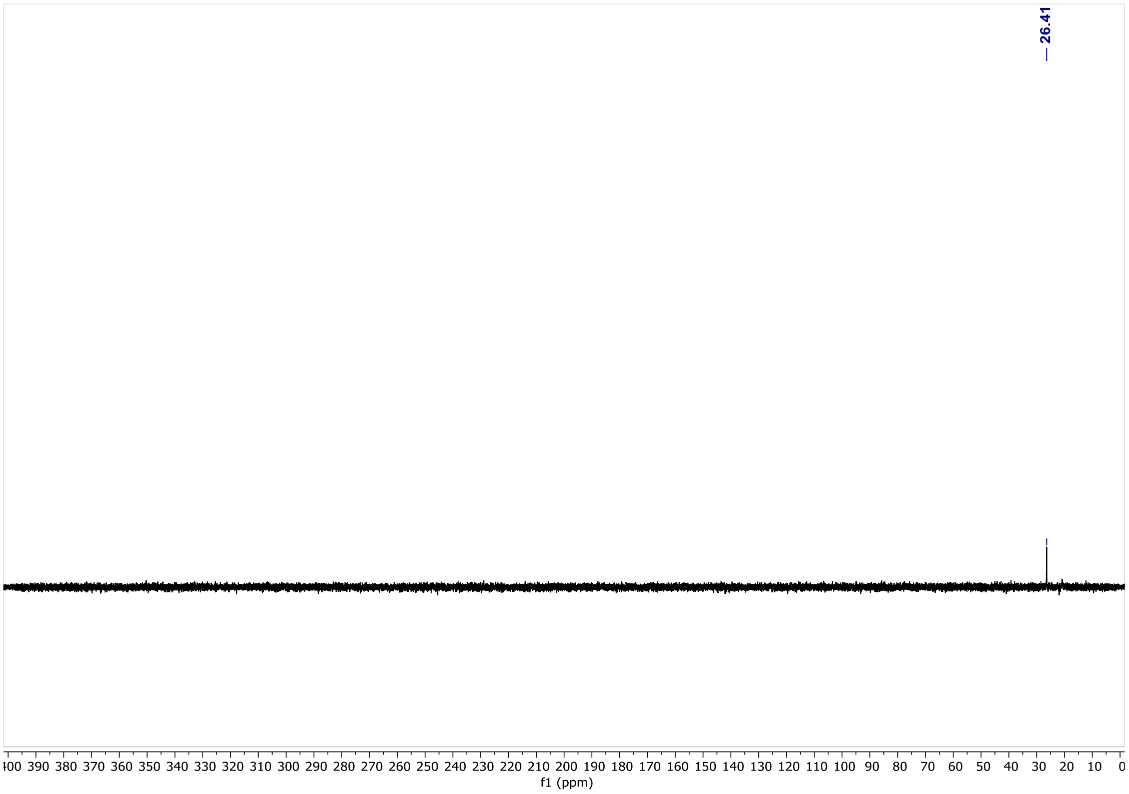

Supplementary Figure 18: The ^{31}P NMR spectrum of **1g** (CD_2Cl_2, 122 MHz, 298 K).

Supplementary Computational Details

Supplementary Note 8: General Considerations

The geometry optimisations for the complexes were done at the M06-2X/def2-TZVP level of theory[14] using the SPARTAN´20 program[15] with dichloromethane (dielectric = 8.82) as a solvent using conductor like polarisable continuum model (C-PCM).[16,17] The initial models of the complexes were built using SPARTAN´20 and optimised at the MM-level before the DFT calculations. The iodine(I) pnictogenate complexes (**1a**-**1g** and **2d**) were built up from the corresponding MM-level optimised iodine(I) pnictogenates so that the N···O intramolecular distance was approximately 4.45 Å and the O–I···N angle approximately 180°, and then optimised without restrictions at the M06-2X/def2-TZVP level of theory.

Supplementary Note 9: Cartesian Coordinates

1a

P	0.574335	-0.121364	1.624626
O	0.089877	0.905369	2.583840
O	1.448729	0.356243	0.433352
I	0.659878	1.697742	-1.066275
C	1.594686	-1.369334	2.439514
C	3.082091	-3.326182	3.744948
C	2.582363	-2.072957	1.752016
C	1.357746	-1.650536	3.784121
C	2.098940	-2.627066	4.434948
C	3.324632	-3.047865	2.404959
H	2.773604	-1.849158	0.709833
H	0.595619	-1.096708	4.319185
H	1.912394	-2.840464	5.479791
H	4.094746	-3.588639	1.869596
H	3.661818	-4.086903	4.252701
C	-0.822150	-1.014148	0.894935
C	-2.977323	-2.388060	-0.208327
C	-2.093565	-0.882540	1.446711
C	-0.634978	-1.840489	-0.213591
C	-1.708606	-2.524871	-0.763538
C	-3.169751	-1.567307	0.894899
H	-2.235133	-0.235164	2.303470
H	0.352449	-1.940544	-0.650610
H	-1.559461	-3.163086	-1.625439
H	-4.157069	-1.459058	1.325597
H	-3.815388	-2.921812	-0.639005
H	-2.668260	3.382449	-4.788257
C	-1.744378	3.638898	-4.289639
C	0.580360	4.195514	-2.967525
C	-1.245247	2.807576	-3.304963
C	-1.039900	4.788806	-4.612515
C	0.140862	5.071882	-3.941357
N	-0.106694	3.090696	-2.666035
H	-1.751273	1.897319	-3.009720
H	0.718488	5.958056	-4.162486
H	1.492812	4.365290	-2.410742
H	-1.407255	5.458416	-5.379017

1b

P	2.079943	0.790287	0.381640
H	-4.650804	0.969196	-4.724878
C	-4.421875	0.761601	-3.687957
C	-3.728002	0.255660	-1.106402
C	-3.104304	0.666162	-3.293488
C	-5.440366	0.596576	-2.750536
C	-5.065427	0.339576	-1.434517
N	-2.770457	0.417445	-2.023791
H	-2.287265	0.791880	-3.992393
H	-5.810321	0.208601	-0.661116
H	-3.399601	0.059951	-0.093553
C	-6.880304	0.671396	-3.151203
H	-7.026793	1.412279	-3.935918
H	-7.512127	0.915667	-2.299309
H	-7.200165	-0.296104	-3.545020
O	1.998497	2.267487	0.519918
O	1.498152	0.150895	-0.908774
I	-0.596164	0.298243	-1.424627
C	1.247610	-0.024176	1.768549
C	-0.011982	-1.265896	3.918694
C	0.880302	-1.367961	1.687514
C	0.977591	0.692371	2.931502
C	0.348305	0.072769	4.004542
C	0.253461	-1.986949	2.759265
H	1.079170	-1.926419	0.779638
H	1.253301	1.738231	2.986696
H	0.135756	0.635320	4.904893
H	-0.030345	-3.029523	2.691669
H	-0.502959	-1.748808	4.754197
C	3.794362	0.215624	0.403063
C	6.443521	-0.634987	0.487771
C	4.145965	-1.034801	-0.103918
C	4.777237	1.036562	0.951050
C	6.099190	0.612468	0.991937
C	5.466661	-1.458503	-0.060382
H	3.387725	-1.673511	-0.541453
H	4.503309	2.010937	1.337161
H	6.859172	1.255988	1.417230
H	5.736111	-2.429128	-0.457738
H	7.473917	-0.966405	0.520044

1c

P	-2.614289	-0.641879	0.542572
H	4.169421	-1.145820	-4.408737
C	3.927302	-0.775508	-3.420906
C	3.203311	0.145818	-0.962161
C	2.607207	-0.713369	-3.024801
C	4.930056	-0.356253	-2.549933
C	4.541459	0.107364	-1.294815
N	2.259775	-0.257407	-1.817793
H	1.799735	-1.027098	-3.674029
H	5.276961	0.447745	-0.577638
H	2.861746	0.506035	-0.000424
O	-2.528391	-2.118976	0.683135
O	-2.016407	0.003021	-0.736945
I	0.083931	-0.150863	-1.234741
C	-1.805238	0.178688	1.939557
C	-0.582079	1.428029	4.107198
C	-1.407841	1.513139	1.849844
C	-1.585586	-0.524127	3.121536
C	-0.975301	0.099144	4.203113
C	-0.797747	2.135345	2.929645
H	-1.568008	2.061726	0.928486
H	-1.886764	-1.562573	3.185923
H	-0.803252	-0.452769	5.118662
H	-0.486772	3.169635	2.853219
H	-0.104641	1.913202	4.949286
C	-4.330179	-0.073581	0.543777
C	-6.981121	0.772901	0.601854
C	-4.671463	1.195873	0.077746
C	-5.323558	-0.915044	1.038487
C	-6.646920	-0.492602	1.065991
C	-5.993056	1.617309	0.107317
H	-3.903288	1.850165	-0.317016
H	-5.055702	-1.903174	1.392112
H	-7.416296	-1.152356	1.446819
H	-6.254568	2.602233	-0.258549
H	-8.012427	1.102167	0.622458
C	6.377893	-0.446538	-2.928403
H	6.477880	-0.333586	-4.008598
H	6.925076	0.367707	-2.452219
C	6.967354	-1.791727	-2.492406
H	6.441575	-2.615597	-2.975977
H	6.878274	-1.916744	-1.412563
H	8.021940	-1.849656	-2.760085

1d

P	0.926054	-1.215793	2.739151
O	0.440342	-0.196588	3.707974
O	1.798316	-0.737891	1.555438
I	0.985173	0.617069	0.018796
C	1.930224	-2.477319	3.558269
C	3.397505	-4.451585	4.862977
C	2.930880	-3.170809	2.879381
C	1.670822	-2.777987	4.894648
C	2.401444	-3.762754	5.545136
C	3.662850	-4.154370	3.531421
H	3.139762	-2.932034	1.843955
H	0.898702	-2.232287	5.423736
H	2.196788	-3.990843	6.583479
H	4.442626	-4.687142	3.002068
H	3.969217	-5.218690	5.370226
C	-0.476881	-2.103091	2.009554
C	-2.639258	-3.462319	0.899206
C	-1.747805	-1.969256	2.561767
C	-0.294418	-2.925069	0.896924
C	-1.371054	-3.602121	0.343568
C	-2.827439	-2.646380	2.006748
H	-1.886373	-1.325307	3.421533
H	0.692349	-3.026654	0.458947
H	-1.224710	-4.236307	-0.521777
H	-3.814173	-2.535636	2.438122
H	-3.479902	-3.989631	0.465646
H	-2.354716	2.230685	-3.654951
C	-1.430739	2.518143	-3.177656
C	0.890352	3.096090	-1.873691
C	-0.920625	1.704918	-2.202101
C	-0.744251	3.704473	-3.537776
C	0.457600	3.968416	-2.835396
N	0.221883	1.977224	-1.552869
H	-1.431084	0.794658	-1.913767
H	1.051197	4.846621	-3.036456
H	1.806063	3.282006	-1.326960
N	-1.205841	4.529806	-4.488340
C	-0.469215	5.736689	-4.829286
H	0.534661	5.496112	-5.185548
H	-0.384317	6.400857	-3.966547
H	-1.000451	6.259241	-5.617756
C	-2.444592	4.221391	-5.185552
H	-2.645090	5.007268	-5.906161
H	-3.283399	4.165408	-4.488335
H	-2.368476	3.270790	-5.717745

1e

P	3.678690	0.514400	1.079108
H	-3.056478	1.314605	-3.953658
C	-2.832476	1.009351	-2.942541
C	-2.120445	0.273996	-0.414924
C	-1.524870	0.872529	-2.565519
C	-3.864187	0.764057	-2.002087
C	-3.453293	0.387570	-0.698993
N	-1.160799	0.508714	-1.324979
H	-0.720331	1.059001	-3.265557
H	-4.172986	0.174737	0.077087
H	-1.783834	-0.018574	0.571710
O	3.607979	1.955942	1.440133
O	3.111202	0.076464	-0.290465
I	0.972946	0.308447	-0.779196
C	2.825678	-0.487799	2.326268
C	1.533453	-2.030060	4.252002
C	2.523201	-1.825892	2.069745
C	2.476278	0.070422	3.552503
C	1.829814	-0.698550	4.513146
C	1.880288	-2.594476	3.028782
H	2.785223	-2.262286	1.112092
H	2.706756	1.110755	3.746391
H	1.557082	-0.258366	5.464151
H	1.645765	-3.631314	2.823469
H	1.030360	-2.629407	5.000605
C	5.386915	-0.080682	1.064840
C	8.013778	-1.001953	1.128946
C	5.762854	-1.183550	0.299028
C	6.335265	0.558131	1.861390
C	7.645347	0.098740	1.893255
C	7.072882	-1.641876	0.330831
H	5.028554	-1.674514	-0.327839
H	6.044189	1.420119	2.449531
H	8.379424	0.600127	2.511412
H	7.360896	-2.496042	-0.268720
H	9.035585	-1.359601	1.153342
N	-5.152583	0.879398	-2.326178
C	-5.633474	1.167224	-3.679508
H	-5.118791	0.540917	-4.408544
H	-5.448145	2.215817	-3.931251
C	-7.125892	0.863039	-3.590132
H	-7.294112	-0.200608	-3.766980
H	-7.703731	1.430004	-4.316918
C	-7.465327	1.211817	-2.141637
H	-7.549225	2.293860	-2.027952
H	-8.389413	0.755487	-1.793347
C	-6.249539	0.707502	-1.370737
H	-6.351156	-0.347761	-1.099453
H	-6.059314	1.280141	-0.462652

1f

P	-1.390456	1.840055	3.670402
O	-0.834660	0.819163	4.598587
O	-2.214610	1.348600	2.457268
I	-1.299122	0.105721	0.888799
C	-2.495476	2.985893	4.528862
C	-4.109530	4.801802	5.889799
C	-3.514992	3.659014	3.857049
C	-2.291903	3.226611	5.886568
C	-3.096277	4.131874	6.565260
C	-4.319775	4.563535	4.536749
H	-3.680812	3.466279	2.804193
H	-1.504637	2.697115	6.409514
H	-2.935055	4.313092	7.620463
H	-5.112979	5.080795	4.012073
H	-4.737777	5.507367	6.419088
C	-0.057081	2.867078	2.995834
C	1.992651	4.446148	1.966421
C	1.223284	2.793376	3.536619
C	-0.305714	3.738625	1.934816
C	0.714433	4.525768	1.422320
C	2.246903	3.580413	3.021760
H	1.413527	2.111229	4.356145
H	-1.300206	3.794341	1.505742
H	0.516789	5.198900	0.597545
H	3.242129	3.516242	3.443413
H	2.789661	5.059397	1.564574
H	0.138596	-0.988115	-3.996912
C	0.146512	-1.432460	-3.015284
C	0.134492	-2.328695	-0.451414
C	-0.407043	-0.709589	-1.991881
C	0.738592	-2.695647	-2.765006
C	0.715793	-3.112920	-1.409794
N	-0.424002	-1.139477	-0.723420
H	-0.847981	0.261603	-2.176670
H	1.122718	-4.059375	-1.093803
H	0.102293	-2.649539	0.581919
N	1.272355	-3.455618	-3.748503
C	1.170420	-4.184326	-6.106367
C	2.202733	-5.657902	-4.398789
C	2.267773	-5.206576	-5.850933
C	2.302273	-4.461496	-3.465555
C	1.256571	-3.007044	-5.142036
H	0.193840	-4.661806	-5.987655
H	1.260951	-6.181973	-4.213754
H	3.244821	-4.752540	-6.045613
H	3.282039	-3.982891	-3.593547
H	2.160676	-2.417310	-5.342215
H	1.223596	-3.793850	-7.123781
H	3.016432	-6.345555	-4.163359
H	2.163413	-6.056729	-6.526310
H	2.230601	-4.788539	-2.435371
H	0.393221	-2.370061	-5.303811

1g

P	-1.240070	1.668145	3.393200
O	-0.678184	0.629869	4.297623
O	-2.057997	1.197967	2.165766
I	-1.154328	-0.062931	0.619881
C	-2.363020	2.778815	4.273807
C	-4.013242	4.534490	5.668613
C	-3.353768	3.493443	3.601745
C	-2.206250	2.947239	5.648305
C	-3.029341	3.822860	6.343992
C	-4.176506	4.368152	4.298113
H	-3.483849	3.356242	2.535239
H	-1.441481	2.384560	6.170136
H	-2.905094	3.948118	7.412200
H	-4.947537	4.917758	3.773077
H	-4.655934	5.216636	6.211098
C	0.083744	2.723980	2.747588
C	2.117740	4.361572	1.779775
C	1.348214	2.689364	3.328859
C	-0.156553	3.585141	1.676516
C	0.856186	4.401249	1.194560
C	2.363868	3.505622	2.845001
H	1.532929	2.014555	4.155778
H	-1.137853	3.610198	1.215622
H	0.665150	5.066185	0.361607
H	3.346640	3.471685	3.298327
H	2.908479	4.998077	1.402499
H	-0.043068	-1.394347	-4.300122
C	0.089019	-1.762230	-3.296192
C	0.356011	-2.477308	-0.688927
C	-0.417822	-1.002933	-2.274371
C	0.761836	-2.975050	-3.017605
C	0.896363	-3.295618	-1.646162
N	-0.299934	-1.347584	-0.985866
H	-0.937252	-0.075438	-2.479311
H	1.417032	-4.176760	-1.309277
H	0.446912	-2.719121	0.362450
N	1.239398	-3.780375	-4.001420
C	1.468842	-4.407358	-6.368641
C	2.295628	-5.861068	-4.784767
O	2.542166	-5.265002	-6.040547
C	2.213376	-4.825178	-3.679973
C	1.333356	-3.273376	-5.371080
H	0.536811	-4.984336	-6.401664
H	1.360326	-6.431937	-4.827851
H	3.197935	-4.370101	-3.520367
H	2.189881	-2.594019	-5.454685
H	1.665442	-3.995229	-7.356783
H	3.117654	-6.543628	-4.576302
H	1.918309	-5.344232	-2.772514
H	0.429837	-2.726763	-5.626949

$Me_2As(O)O$–I–(DMAP)

As	1.077761	-3.005083	4.050534
O	0.636180	-1.735425	4.998682
O	1.986308	-2.535606	2.660050
I	1.193535	-1.147649	1.263524
H	-2.183745	0.641942	-2.390157
C	-1.251639	0.907028	-1.915524
C	1.084007	1.426360	-0.618466
C	-0.745655	0.074693	-0.951011
C	-0.546843	2.083359	-2.266987
C	0.662008	2.319563	-1.568756
N	0.402467	0.316504	-0.304744
H	-1.270603	-0.831072	-0.672542
H	1.267740	3.191089	-1.763691
H	2.006700	1.591867	-0.075617
N	-0.998902	2.926692	-3.210260
C	-0.242740	4.123237	-3.541477
H	0.757591	3.868589	-3.898632
H	-0.146333	4.780475	-2.674337
H	-0.763618	4.660823	-4.327112
C	-2.247034	2.646076	-3.900401
H	-2.435002	3.435840	-4.620549
H	-3.084025	2.607099	-3.199490
H	-2.194903	1.694124	-4.433463
C	2.250050	-4.241902	4.928790
H	3.143566	-3.702224	5.237281
H	2.512923	-5.041140	4.238143
H	1.741685	-4.649589	5.801012
C	-0.441015	-3.971631	3.380856
H	-0.100861	-4.761669	2.713390
H	-1.080815	-3.275082	2.840864
H	-0.988785	-4.397286	4.220092

(*S*-BINOL)P(O)O–I–(py)

H	0.730668	0.315816	-3.235388
C	-0.156571	0.752144	-2.795657
H	-1.002193	1.437840	-4.624443
C	-1.108318	1.371848	-3.548469
C	-1.428855	1.172691	-0.750688
C	-2.237666	1.968498	-2.934475
C	-0.327460	0.671141	-1.400884
C	-2.395841	1.889946	-1.525177
C	-3.198619	2.667555	-3.706432
H	-3.606801	2.557641	0.140700
C	-4.258974	3.289055	-3.110155
H	-3.065586	2.706220	-4.781303
H	-4.986216	3.825160	-3.706365
C	-4.396198	3.247766	-1.705410
H	-5.222519	3.764104	-1.233605
C	-3.492743	2.569257	-0.934966
H	-5.613501	-1.194042	0.597302
C	-4.819582	-0.762827	1.193973
H	-3.697035	-0.161288	-0.504263
C	-3.746619	-0.181553	0.576111
C	-3.883493	-0.303253	3.358438
C	-2.687464	0.383979	1.331870
C	-4.899637	-0.814720	2.602706
C	-2.751798	0.292348	2.747596
C	-1.546989	0.995396	0.718371
H	-5.758606	-1.272055	3.076683
H	-1.735049	0.696906	4.609756
H	-3.917535	-0.352541	4.440549
C	-0.508800	1.379067	1.532668
C	-0.559281	1.284487	2.936127
H	0.292405	1.627462	3.508713
C	-1.671800	0.769773	3.530855
O	0.658438	0.034352	-0.669080
O	0.638596	1.914937	0.974869
P	1.663255	0.951242	0.198124
O	2.563591	1.765736	-0.625746
O	2.247025	-0.010923	1.217249
I	3.973486	-1.337489	0.792278
H	5.285608	-2.728811	-1.601804
C	5.948295	-3.087675	-0.825505
C	7.534545	-3.910799	1.241918
C	7.011881	-3.926175	-1.094041
N	5.686725	-2.679890	0.420665
C	6.455383	-3.073569	1.442035
C	7.816774	-4.344738	-0.044768
H	7.196842	-4.240340	-2.111372
H	6.185751	-2.703548	2.422258
H	8.655386	-5.003177	-0.228322
H	8.137095	-4.212952	2.086499

Supplementary References

1. Moodley, V., Mthethwa, L., Pillay, M. N., Omondi, B. & van Zyl, W. E. The silver(I) coordination polymer $[AgO_2PPh_2]_n$ and unsupported Ag···Ag interactions derived from aminophosphinate and phosphinic acid. *Polyhedron* **99**, 87–95 (2015).

2. Sheldrick, G. M. SHELXT – Integrated space-group and crystal-structure determination. *Acta Crystallogr. Sect. A Found. Adv.* **71**, 3–8 (2015).

3. Dolomanov, O. V., Bourhis, L. J., Gildea, R. J., Howard, J. A. K. & Puschmann, H. OLEX2 : a complete structure solution, refinement and analysis program. *J. Appl. Crystallogr.* **42**, 339–341 (2009).

4. Sheldrick, G. M. Crystal structure refinement with SHELXL. *Acta Crystallogr. Sect. C, Struct. Chem.* **71**, 3–8 (2015).

5. Campos, P. J., Arranz, J. & Rodríguez, M. A. α-Iodination of enaminones with *bis*(pyridine)iodonium(I) tetrafluoroborate. *Tetrahedron Lett.* **38**, 8397–8400 (1997).

6. Wilson, L. M. E., Rissanen, K. & Ward, J. S. Iodination of antipyrine with $[N–I–N]^+$ and carbonyl hypoiodite iodine(I) complexes. *New J. Chem.* **47**, 2978–2982 (2023).

7. Schumacher, C. *et al.* Halogen bonding and mechanochemistry combined: synthesis, characterization, and application of N-iodosaccharin pyridine complexes. *Org. Chem. Front.* **11**, 781–795 (2024).

8. Ward, J. S. *et al.* Carbonyl hypoiodites from pivalic and trimesic acid and their silver(i) intermediates. *Dalton Trans.* **51**, 14646–14653 (2022).

9. Kramer, E., Yu, S., Ward, J. S. & Rissanen, K. Dihypoiodites stabilised by 4-ethylpyridine through O–I–N halogen bonds. *Dalton Trans.* **50**, 14990–14993 (2021).

10. Ward, J. S., Fiorini, G., Frontera, A. & Rissanen, K. Asymmetric $[N–I–N]^+$ halonium complexes. *Chem. Commun.* **56**, 8428–8431 (2020).

11. Mattila, M., Rissanen, K. & Ward, J. S. Chiral carbonyl hypoiodites. *Chem. Commun.* **59**, 4648–4651 (2023).

12. Yu, S., Ward, J. S., Truong, K.-N. & Rissanen, K. Carbonyl Hypoiodites as Extremely Strong Halogen Bond Donors. *Angew. Chem. Int. Ed.* **60**, 20739–20743 (2021).

13. Puttreddy, R., Kumar, P. & Rissanen, K. Pyridine Iodine(I) Cations: Kinetic Trapping as a Sulfonate Complexes. *Chem. – A Eur. J.* e202304178 (2024).

14. Zhao, Y. & Truhlar, D. G. The M06 suite of density functionals for main group thermochemistry, thermochemical kinetics, noncovalent interactions, excited states, and transition elements: two new functionals and systematic testing of four M06-class functionals and 12 other function. *Theor. Chem. Acc.* **120**, 215–241 (2008).

15. Spartan’20. Spartan’20, Wavefunction Inc., Irvine CA, USA 2018

16. Zhang, X. & Herbert, J. M. Excited-State Deactivation Pathways in Uracil versus Hydrated Uracil: Solvatochromatic Shift in the 1nπ* State is the Key. *J. Phys. Chem. B* **118**, 7806–7817 (2014).

17. Lange, A. W. & Herbert, J. M. Symmetric versus asymmetric discretization of the integral equations in polarizable continuum solvation models. *Chem. Phys. Lett.* **509**, 77–87 (2011).
