# Supplementary material for: Iodine(I) pnictogenate complexes as Iodination reagents
Source: Commun Chem. 2024 Jul 17;7:159. doi: 10.1038/s42004-024-01240-0 (PMC11255316; doi:10.1038/s42004-024-01240-0)
Supplement: Supplementary file 2 — Description of Additional Supplementary Files [file 42004_2024_1240_MOESM2_ESM.pdf]

Description of Additional Supplementary Files

File name: Supplementary Data 1

Description: Combined CIFs of all new solid-state data reported.

File name: Supplementary Data 2

Description: Combined Checkcif reports of all new solid-state data reported in .cif format.
