# Supplementary material for: Iodine(I) pnictogenate complexes as Iodination reagents
Source: Commun Chem. 2024 Jul 17;7:159. doi: 10.1038/s42004-024-01240-0 (PMC11255316; doi:10.1038/s42004-024-01240-0)

checkCIF/PLATON report

You have not supplied any structure factors. As a result the full set of tests cannot be run.

THIS REPORT IS FOR GUIDANCE ONLY. IF USED AS PART OF A REVIEW PROCEDURE FOR PUBLICATION, IT SHOULD NOT REPLACE THE EXPERTISE OF AN EXPERIENCED CRYSTALLOGRAPHIC REFEREE.

No syntax errors found. CIF dictionary Interpreting this report

Datablock: 1b

No errors found in this datablock

```
Bond precision:   C-C = 0.0048 A           Wavelength=1.54184

Cell:           a=8.9987(2)        b=9.4164(3)       c=11.8309(5)
                alpha=72.528(3)    beta=76.407(3)    gamma=80.613(2)
Temperature:    120 K
```

	Calculated	Reported
Volume	924.82(6)	924.82(6)
Space group	P -1	P -1
Hall group	-P 1	-P 1
Moiety formula	C18 H17 I N O2 P, H2 O	C18 H17 I N O2 P, H2 O
Sum formula	C18 H19 I N O3 P	C18 H19 I N O3 P
Mr	455.21	455.21
Dx,g cm-3	1.635	1.635
Z	2	2
Mu (mm-1)	14.553	14.553
F000	452.0	452.0
F000'	453.15	
h,k,lmax	11,11,14	11,11,14
Nref	3790	3779
Tmin,Tmax		0.578,0.610
Tmin'		

```
Correction method= # Reported T Limits: Tmin=0.578 Tmax=0.610
AbsCorr = SPHERE

Data completeness= 0.997           Theta(max)= 74.491

R(reflections)= 0.0268( 3464)                wR2(reflections)=
                                             0.0673( 3779)
S = 1.047                    Npar= 224
```

Datablock: 1c

Bond precision:	C-C = 0.0046 A		Wavelength=1.54184
Cell:	a=9.0653(2)	b=9.2711(2)	c=12.0375(2)
	alpha=75.857(1)	beta=84.020(2)	gamma=87.137(2)
Temperature:	120 K		

	Calculated	Reported
Volume	975.38(3)	975.38(3)
Space group	P -1	P -1
Hall group	-P 1	-P 1
Moiety formula	C19 H19 I N O2 P, H2 O	C19 H19 I N O2 P, H2 O
Sum formula	C19 H21 I N O3 P	C19 H21 I N O3 P
Mr	469.24	469.24
Dx,g cm-3	1.598	1.598
Z	2	2
Mu (mm-1)	13.818	13.818
F000	468.0	468.0
F000'	469.18	
h,k,lmax	11,11,15	11,11,15
Nref	3994	3983
Tmin,Tmax	0.443,0.759	0.413,0.769
Tmin'	0.200	

Correction method= # Reported T Limits: Tmin=0.413 Tmax=0.769
AbsCorr = ANALYTICAL

Data completeness= 0.997 Theta(max)= 74.471

R(reflections)= 0.0279(3667) wR2(reflections)= 0.0725(3983)

S = 1.061 Npar= 253

The following ALERTS were generated. Each ALERT has the format
test-name_ALERT_alert-type_alert-level.
Click on the hyperlinks for more details of the test.

Alert level C

PLAT230_ALERT_2_C Hirshfeld Test Diff for I1 --O11 . 6.8 s.u.

Alert level G

```
PLAT301_ALERT_3_G Main Residue  Disorder .............(Resd    1)         8% Note
```

```
   0 ALERT level A = Most likely a serious problem - resolve or explain
   0 ALERT level B = A potentially serious problem, consider carefully
   1 ALERT level C = Check. Ensure it is not caused by an omission or oversight
   1 ALERT level G = General information/check it is not something unexpected

   0 ALERT type 1 CIF construction/syntax error, inconsistent or missing data
   1 ALERT type 2 Indicator that the structure model may be wrong or deficient
   1 ALERT type 3 Indicator that the structure quality may be low
   0 ALERT type 4 Improvement, methodology, query or suggestion
   0 ALERT type 5 Informative message, check
```

Datablock: 1d

Bond precision: C-C = 0.0049 A Wavelength=1.54184

Cell:	a=7.3598(2)	b=9.2439(2)	c=15.5137(6)
	alpha=88.624(3)	beta=78.608(3)	gamma=75.919(2)
Temperature:	120 K		

	Calculated	Reported
Volume	1003.25(5)	1003.24(5)
Space group	P -1	P -1
Hall group	-P 1	-P 1
Moiety formula	C19 H20 I N2 O2 P [+ solvent]	C19 H20 I N2 O2 P
Sum formula	C19 H20 I N2 O2 P [+ solvent]	C19 H20 I N2 O2 P
Mr	466.24	466.24
Dx,g cm-3	1.543	1.543
Z	2	2
Mu (mm-1)	13.407	13.408
F000	464.0	464.0
F000’	465.14	
h,k,lmax	9,11,19	9,11,19
Nref	4104	4088
Tmin,Tmax	0.561,0.786	0.211,0.795
Tmin’	0.080	

Correction method= # Reported T Limits: Tmin=0.211 Tmax=0.795
AbsCorr = ANALYTICAL

Data completeness= 0.996 Theta(max)= 74.504

R(reflections)= 0.0259(3813) wR2(reflections)= 0.0655(4088)

S = 1.049 Npar= 228

The following ALERTS were generated. Each ALERT has the format
test-name_ALERT_alert-type_alert-level.
Click on the hyperlinks for more details of the test.

Alert level C

PLAT220_ALERT_2_C NonSolvent Resd 1 C Ueq(max)/Ueq(min) Range 3.2 Ratio

---

Alert level G

PLAT003_ALERT_2_G Number of Uiso or Uij Restrained non-H Atoms ... 6 Report
PLAT178_ALERT_4_G The CIF-Embedded .res File Contains SIMU Records 1 Report
PLAT188_ALERT_3_G A Non-default SIMU Restraint Value has been used 0.0200 Report
PLAT605_ALERT_4_G Largest Solvent Accessible VOID in the Structure 88 A**3
PLAT860_ALERT_3_G Number of Least-Squares Restraints .... 36 Note
PLAT869_ALERT_4_G ALERTS Related to the Use of SQUEEZE Suppressed ! Info

---

0 **ALERT level A** = Most likely a serious problem - resolve or explain
0 **ALERT level B** = A potentially serious problem, consider carefully
1 **ALERT level C** = Check. Ensure it is not caused by an omission or oversight
6 **ALERT level G** = General information/check it is not something unexpected

0 ALERT type 1 CIF construction/syntax error, inconsistent or missing data
2 ALERT type 2 Indicator that the structure model may be wrong or deficient
2 ALERT type 3 Indicator that the structure quality may be low
3 ALERT type 4 Improvement, methodology, query or suggestion
0 ALERT type 5 Informative message, check

Datablock: 1e

No errors found in this datablock

Bond precision: C-C = 0.0057 A Wavelength=1.54184

Cell:	a=11.4630(2)	b=9.8435(1)	c=18.8688(3)
	alpha=90	beta=107.360(2)	gamma=90
Temperature:	120 K		

	Calculated	Reported
Volume	2032.10(6)	2032.10(6)
Space group	P 21/n	P 1 21/n 1
Hall group	-P 2yn	-P 2yn
Moiety formula	C21 H22 I N2 O2 P	C21 H22 I N2 O2 P
Sum formula	C21 H22 I N2 O2 P	C21 H22 I N2 O2 P
Mr	492.28	492.27
Dx,g cm-3	1.609	1.609
Z	4	4
Mu (mm-1)	13.274	13.274
F000	984.0	984.0
F000'	986.39	
h,k,lmax	14,12,23	14,12,23
Nref	4162	4155
Tmin,Tmax	0.182,0.287	0.262,0.491
Tmin'	0.075	

Correction method= # Reported T Limits: Tmin=0.262 Tmax=0.491
AbsCorr = ANALYTICAL

Data completeness= 0.998 Theta(max)= 74.502

R(reflections)= 0.0352(3995) wR2(reflections)= 0.0929(4155)

S = 1.077 Npar= 244

Datablock: 1f

Bond precision: C-C = 0.0057 A Wavelength=1.54184

Cell:	a=9.1510(1)	b=23.2007(3)	c=10.0876(1)
	alpha=90	beta=94.492(1)	gamma=90
Temperature:	120 K		

	Calculated	Reported
Volume	2135.12(4)	2135.12(4)
Space group	P 21/n	P 1 21/n 1
Hall group	-P 2yn	-P 2yn
Moiety formula	C22 H24 I N2 O2 P	C22 H24 I N2 O2 P
Sum formula	C22 H24 I N2 O2 P	C22 H24 I N2 O2 P
Mr	506.30	506.30
Dx,g cm-3	1.575	1.575
Z	4	4
Mu (mm-1)	12.651	12.651
F000	1016.0	1016.0
F000'	1018.44	
h,k,lmax	11,28,12	11,29,12
Nref	4373	4372
Tmin,Tmax	0.268,0.462	0.299,0.703
Tmin'	0.103	

Correction method= # Reported T Limits: Tmin=0.299 Tmax=0.703
AbsCorr = GAUSSIAN

Data completeness= 1.000 Theta(max)= 74.483

R(reflections)= 0.0347(4095) wR2(reflections)= 0.0980(4372)

S = 1.062 Npar= 253

The following ALERTS were generated. Each ALERT has the format
test-name_ALERT_alert-type_alert-level.
Click on the hyperlinks for more details of the test.

Alert level C

```
PLAT410_ALERT_2_C Short Intra H...H Contact  H3       ..H8A      .       1.94 Ang.
                                                      x,y,z  =      1_555 Check
PLAT410_ALERT_2_C Short Intra H...H Contact  H5       ..H12B     .       1.93 Ang.
                                                      x,y,z  =      1_555 Check
```

Alert level G

```
PLAT083_ALERT_2_G SHELXL Second Parameter in WGHT  Unusually Large       5.24 Why ?
PLAT143_ALERT_4_G s.u. on c - Axis Small or Missing .............    0.00010 Ang.
```

---

0 **ALERT level A** = Most likely a serious problem - resolve or explain
0 **ALERT level B** = A potentially serious problem, consider carefully
2 **ALERT level C** = Check. Ensure it is not caused by an omission or oversight
2 **ALERT level G** = General information/check it is not something unexpected

0 ALERT type 1 CIF construction/syntax error, inconsistent or missing data

```
3 ALERT type 2 Indicator that the structure model may be wrong or deficient
0 ALERT type 3 Indicator that the structure quality may be low
1 ALERT type 4 Improvement, methodology, query or suggestion
0 ALERT type 5 Informative message, check
```

Datablock: 1g

Bond precision:	C-C = 0.0078 A	Wavelength=1.54184	
Cell:	a=12.4883(4)	b=18.8866(4)	c=9.2728(3)
	alpha=90	beta=98.864(3)	gamma=90
Temperature:	120 K		

	Calculated	Reported
Volume	2160.98(11)	2160.98(11)
Space group	P 21/c	P 1 21/c 1
Hall group	-P 2ybc	-P 2ybc
Moiety formula	C21 H22 I N2 O3 P, H2 O	C21 H22 I N2 O3 P, H2 O
Sum formula	C21 H24 I N2 O4 P	C21 H24 I N2 O4 P
Mr	526.29	526.29
Dx,g cm-3	1.618	1.618
Z	4	4
Mu (mm-1)	12.595	12.595
F000	1056.0	1056.0
F000’	1058.75	
h,k,lmax	15,23,11	15,23,11
Nref	4423	4421
Tmin,Tmax	0.677,0.838	0.457,0.867
Tmin’	0.335	

Correction method= # Reported T Limits: Tmin=0.457 Tmax=0.867
AbsCorr = GAUSSIAN

Data completeness= 1.000 Theta(max)= 74.496

R(reflections)= 0.0417(3442) wR2(reflections)= 0.1062(4421)

S = 1.046 Npar= 268

The following ALERTS were generated. Each ALERT has the format
test-name_ALERT_alert-type_alert-level.
Click on the hyperlinks for more details of the test.

Alert level C

```
PLAT250_ALERT_2_C Large U3/U1 Ratio for <U(i,j)> Tensor(Resd    1)        2.1 Note
```

Alert level G

```
PLAT398_ALERT_2_G Deviating  C-O-C   Angle From 120 for O10      .      108.8 Degree
```

```
   0 ALERT level A = Most likely a serious problem - resolve or explain
   0 ALERT level B = A potentially serious problem, consider carefully
   1 ALERT level C = Check. Ensure it is not caused by an omission or oversight
   1 ALERT level G = General information/check it is not something unexpected

   0 ALERT type 1 CIF construction/syntax error, inconsistent or missing data
   2 ALERT type 2 Indicator that the structure model may be wrong or deficient
   0 ALERT type 3 Indicator that the structure quality may be low
   0 ALERT type 4 Improvement, methodology, query or suggestion
   0 ALERT type 5 Informative message, check
```

It is advisable to attempt to resolve as many as possible of the alerts in all categories. Often the minor alerts point to easily fixed oversights, errors and omissions in your CIF or refinement strategy, so attention to these fine details can be worthwhile. In order to resolve some of the more serious problems it may be necessary to carry out additional measurements or structure refinements. However, the purpose of your study may justify the reported deviations and the more serious of these should normally be commented upon in the discussion or experimental section of a paper or in the "special_details" fields of the CIF. checkCIF was carefully designed to identify outliers and unusual parameters, but every test has its limitations and alerts that are not important in a particular case may appear. Conversely, the absence of alerts does not guarantee there are no aspects of the results needing attention. It is up to the individual to critically assess their own results and, if necessary, seek expert advice.

Publication of your CIF in IUCr journals

A basic structural check has been run on your CIF. These basic checks will be run on all CIFs submitted for publication in IUCr journals (*Acta Crystallographica*, *Journal of Applied Crystallography*, *Journal of Synchrotron Radiation*); however, if you intend to submit to *Acta Crystallographica Section C* or *E* or *IUCrData*, you should make sure that full publication checks are run on the final version of your CIF prior to submission.

Publication of your CIF in other journals

Please refer to the *Notes for Authors* of the relevant journal for any special instructions relating to CIF submission.

PLATON version of 06/01/2024; check.def file version of 05/01/2024

Datablock 1b - ellipsoid plot

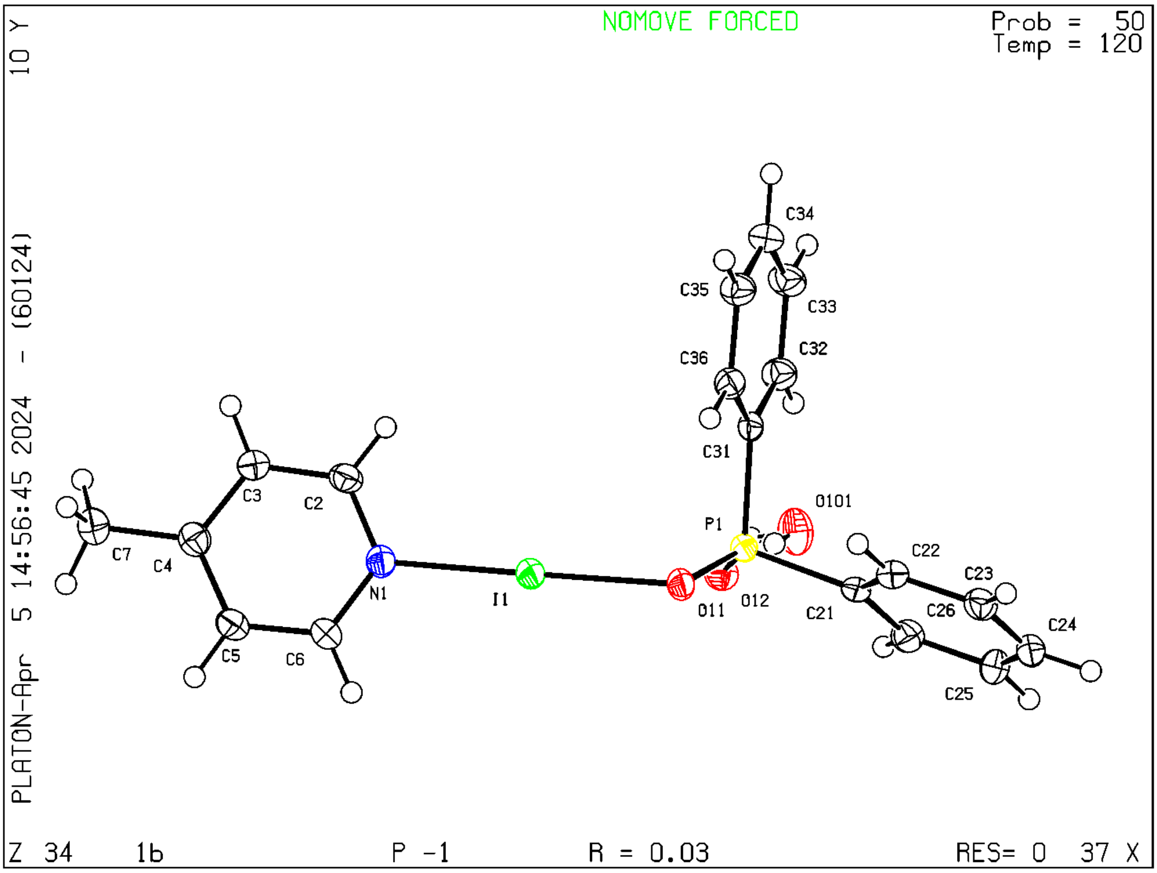

Datablock 1c - ellipsoid plot

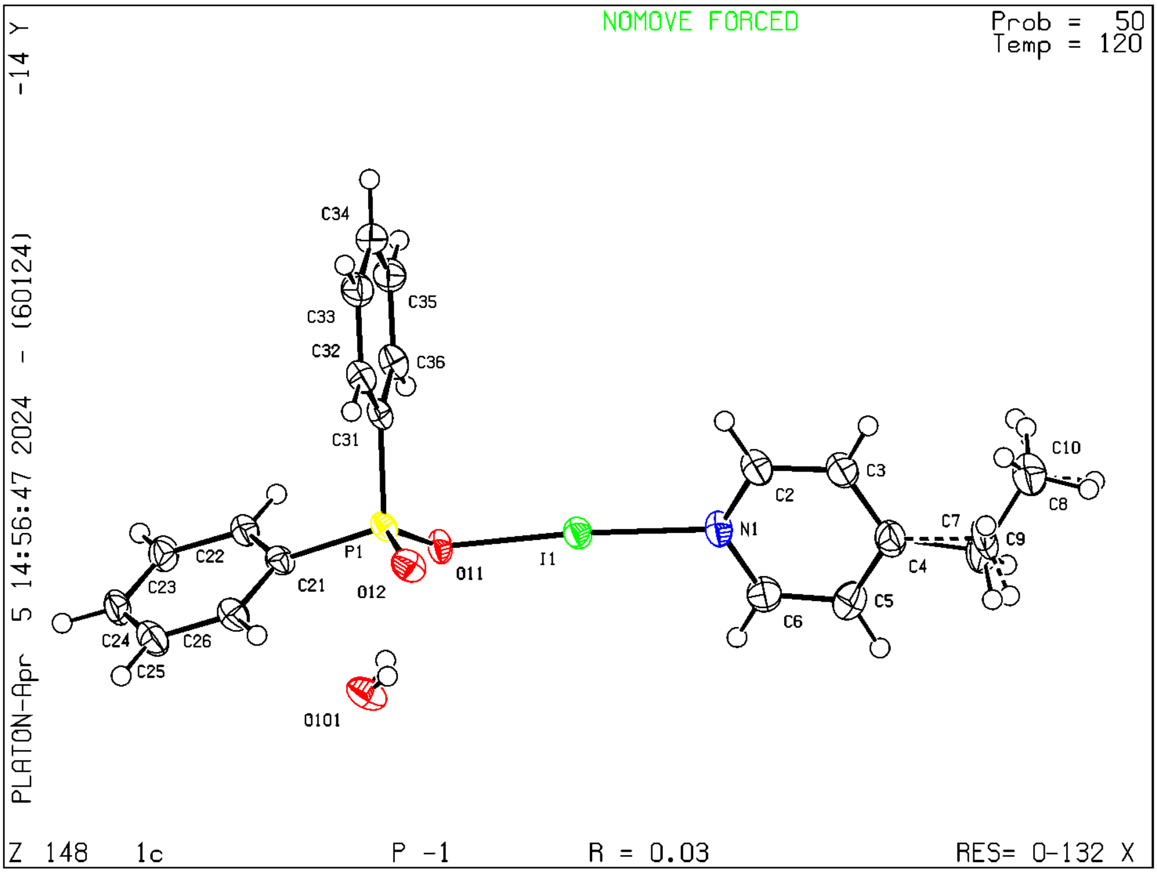

Datablock 1d - ellipsoid plot

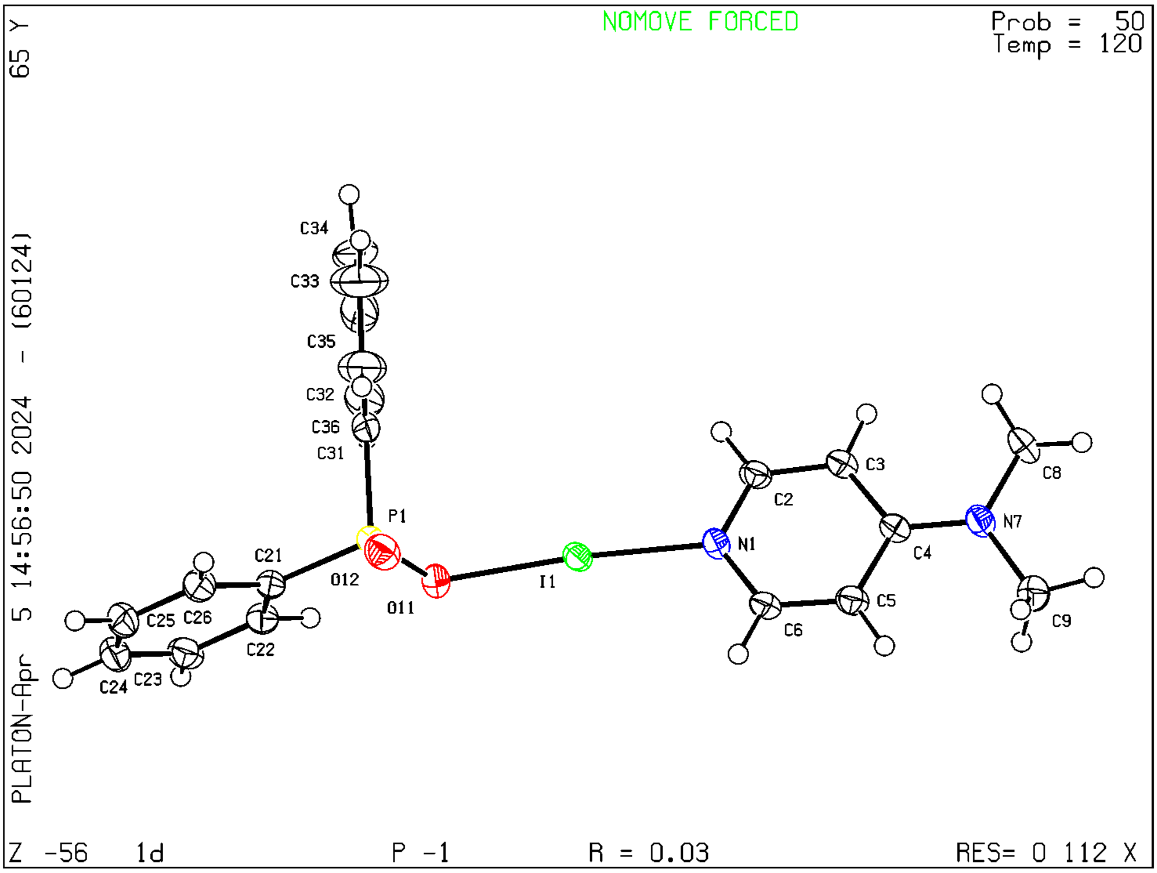

Datablock 1e - ellipsoid plot

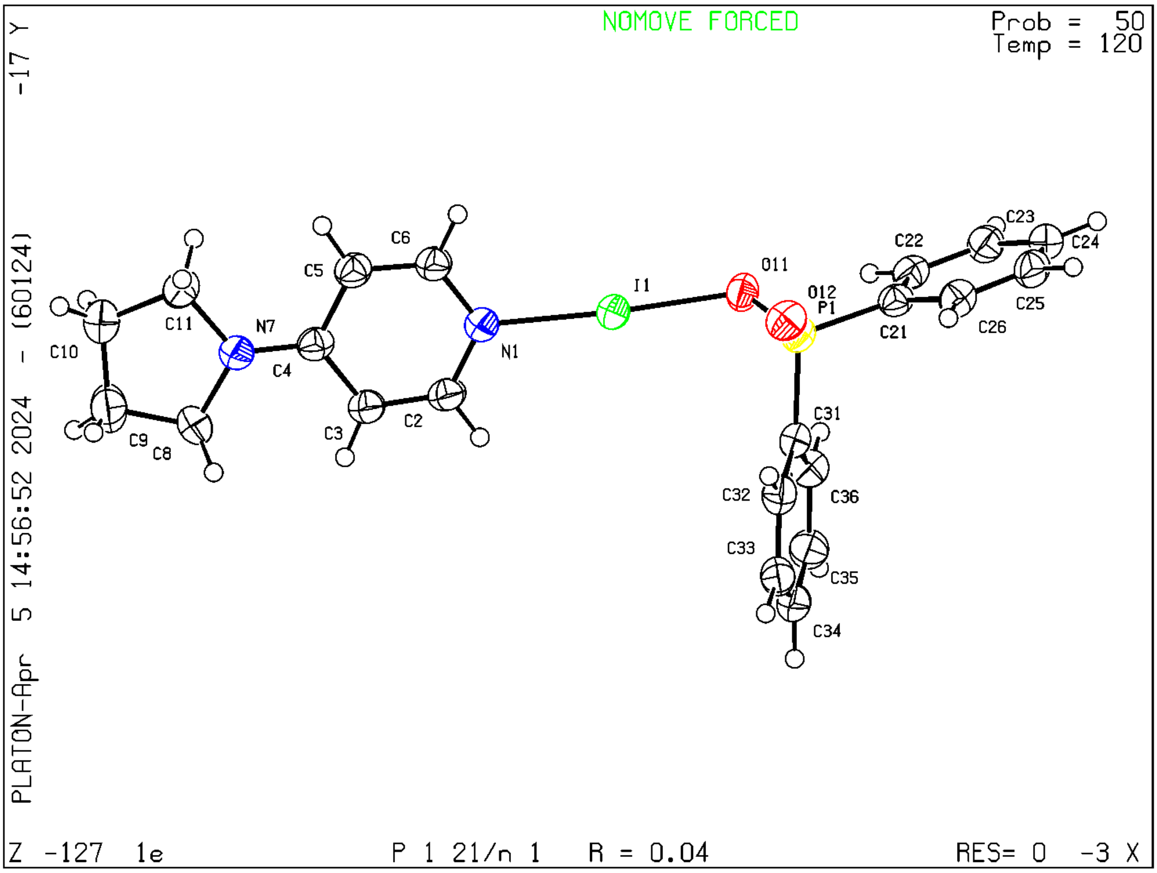

Datablock 1f - ellipsoid plot

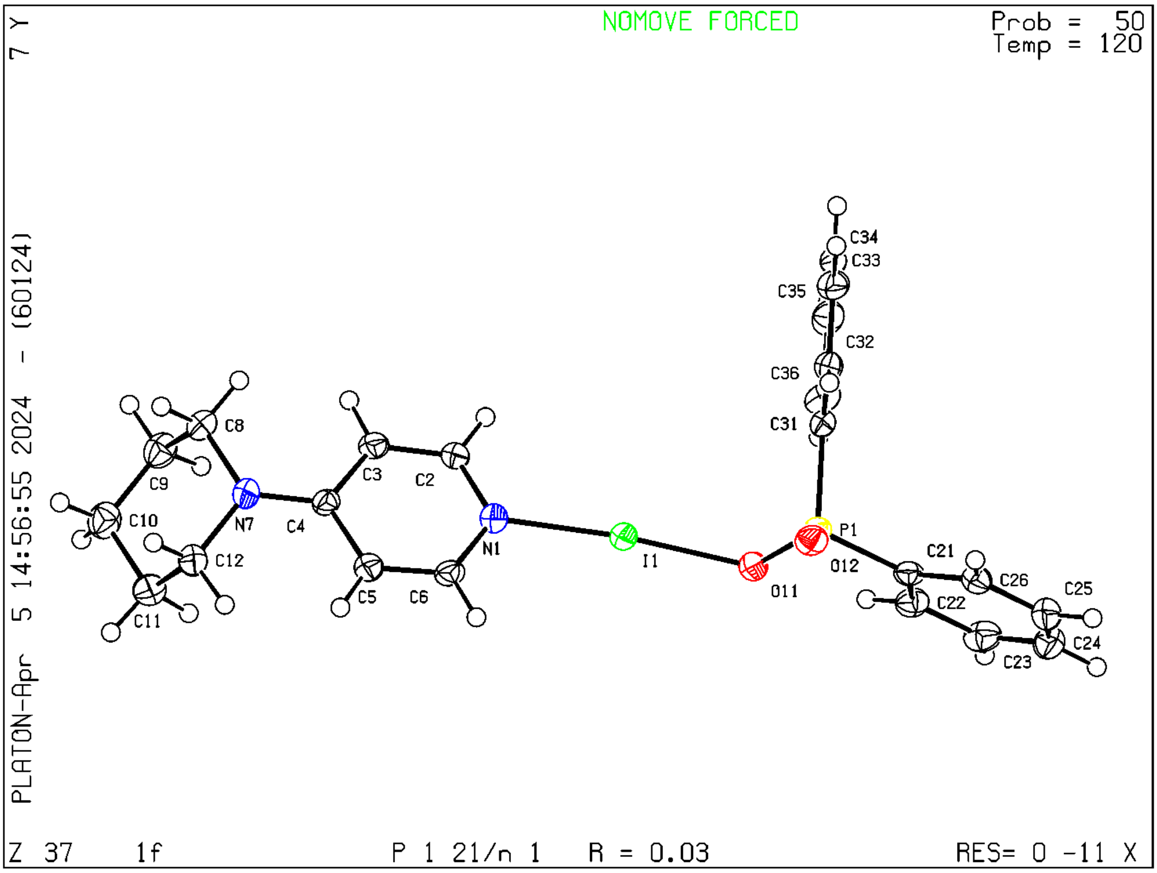

Datablock 1g - ellipsoid plot

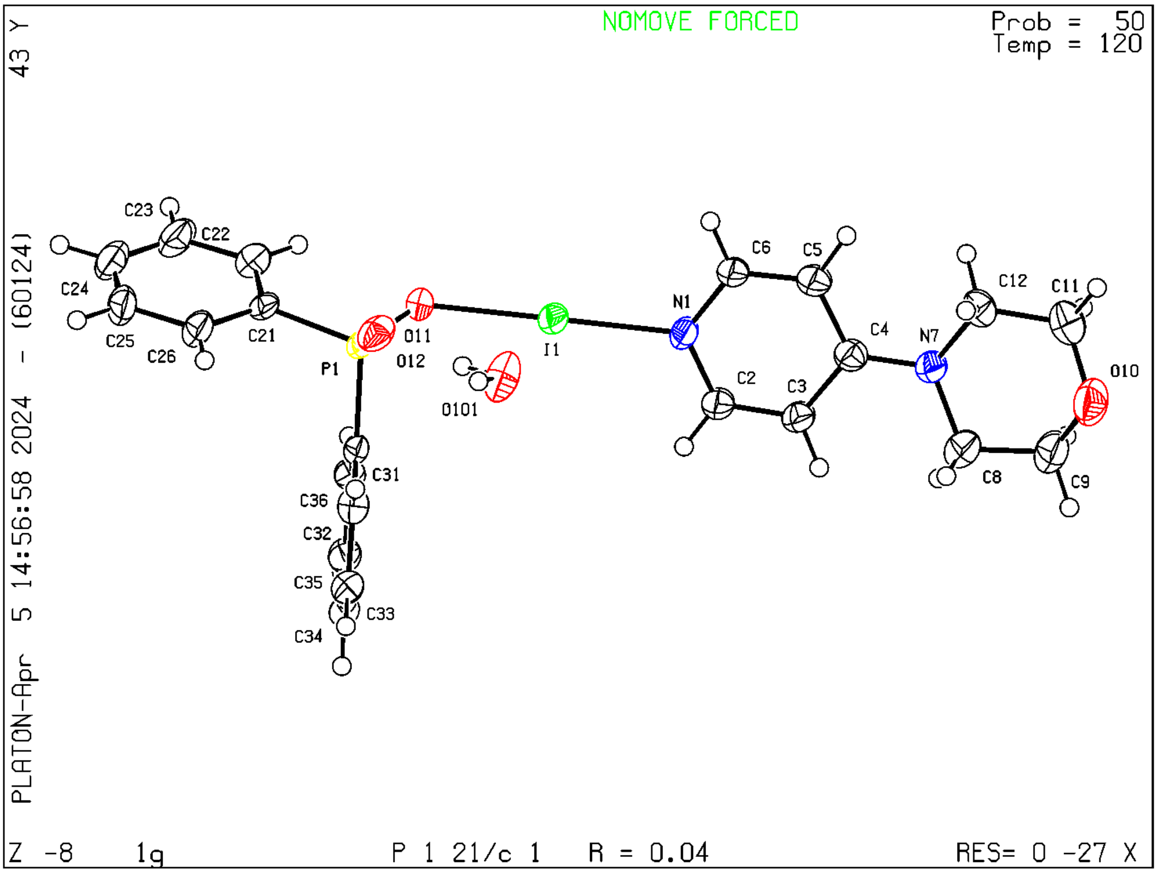

Supplement: Supplementary file 4 — Supplementary Data 2 [file 42004_2024_1240_MOESM4_ESM.pdf]
